# A DNA aptamer recognising a malaria protein biomarker can function as part of a DNA origami assembly

**DOI:** 10.1038/srep21266

**Published:** 2016-02-19

**Authors:** Maia Godonoga, Ting-Yu Lin, Azusa Oshima, Koji Sumitomo, Marco S. L. Tang, Yee-Wai Cheung, Andrew B. Kinghorn, Roderick M. Dirkzwager, Cunshan Zhou, Akinori Kuzuya, Julian A. Tanner, Jonathan G. Heddle

**Affiliations:** 1 Heddle Initiative Research Unit, RIKEN, Saitama, 351-0198, Japan; 2 Department of Life Science and Medical Bioscience, Waseda University, 2-2 Wakamatsu-cho, Shinjuku, Tokyo 162-8480, Japan; 3 NTT Basic Research Laboratories, NTT Corporation, 3-1 Morinosato Wakamiya, Atsugi, Kanagawa 243-0198, Japan; 4 School of Biomedical Sciences, Li Ka Shing Faculty of Medicine, The University of Hong Kong, Pokfulam, Hong Kong SAR, China; 5 School of Food and Biological Engineering, Jiangsu University, No. 301 Xuefu Road, Zhenjiang 212013, China; 6 Department of Chem. Mater. Eng., Kansai University, 3-3-35 Yamate, Suita, Osaka 564-8680, Japan; 7 Malopolska Centre of Biotechnology, Jagiellonian University, Gronostajowa 7, 30–387, Krakow, Poland

## Abstract

DNA aptamers have potential for disease diagnosis and as therapeutics, particularly when interfaced with programmable molecular technology. Here we have combined DNA aptamers specific for the malaria biomarker *Plasmodium falciparum* lactate dehydrogenase (*Pf*LDH) with a DNA origami scaffold. Twelve aptamers that recognise *Pf*LDH were integrated into a rectangular DNA origami and atomic force microscopy demonstrated that the incorporated aptamers preserve their ability to specifically bind target protein. Captured *Pf*LDH retained enzymatic activity and protein-aptamer binding was observed dynamically using high-speed AFM. This work demonstrates the ability of DNA aptamers to recognise a malaria biomarker whilst being integrated within a supramolecular DNA scaffold, opening new possibilities for malaria diagnostic approaches based on DNA nanotechnology.

Aptamers, the nucleic acid equivalent of antibodies, are synthetic single stranded DNA or RNA oligonucleotides capable of specific, high affinity binding to a target. This capability is attributed to their folding into particular three-dimensional configurations exploiting intermolecular binding forces such as hydrogen bonds, electrostatic forces and Van Der Waals forces. Aptamers were first developed in 1990 as short RNA strands with specific binding affinities via the systematic evolution of ligands by exponential enrichment (SELEX) technique^1^,^2^. Subsequent development has also allowed the production of DNA aptamers. Aptamers have had some success as therapeutic agents^3^ and are increasingly targeted for biosensing applications thanks to their advantages in size, stability, reproducibility and cost, especially when compared with antibodies^4^. For sensing applications, aptamers can be labeled with a number of useful reporter molecules such as fluorophores, gold nanoparticles, quantum dots and molecular beacons^5^. The fact that aptamers can be regenerated through simple denaturation and renaturation steps also makes DNA an ideal material for reusable sensors^6^. Aptamers have been integrated into larger scale DNA structures including aptamers for single chain antibodies^7^ and thrombin^8^ (for which the high resolution structure is known)^9^.

Recently a diagnostic aptamer (utilised in this study) was developed to detect the malaria biomarker *Plasmodium falciparum* lactate dehydrogenase (*Pf*LDH). The crystal structure of the aptamer-protein complex was solved at 2.1 Å resolution and revealed two aptamers binding per tetrameric *Pf*LDH^10^ (Fig. 1a). The aptamer was found to bind with a unique distorted hairpin structure comprising a B-helical stem, an asymmetric internal loop and an apical loop. Clear electron density was observed for the first 27 bases in the asymmetric unit of both aptamers, while the last eight bases at the 3′ end displayed no electron density beyond the hairpin structure, an effect ascribed to the distortion of the aptamer tail^10^. The complex interaction was attributed to the extensive salt bridges between each aptamer and the cofactor binding sites of protein subunits^10^. The aptamer was subsequently demonstrated as being capable of detecting *Pf*LDH in blood samples^11^. It is hoped that these aptamers can be developed as useful actuators: components of “smart” malaria diagnostic tools. To this end, integration of the aptamers into DNA origami is being investigated.

The DNA origami method introduced by Rothemund^12^ revolutionised the field of DNA nanotechnology and has allowed for exquisite nano-architectures to be achieved with relative experimental simplicity. The method utilises M13 bacteriophage single-stranded genomic DNA as a template strand, which can be molded into a required 2D or 3D shape using approximately 200 complementary “staple” strands. Functionally, DNA origami can form the basis of single molecule analysis systems^13^ and has potential as a way of arraying enzymes, which may be advantageous for detector systems. This has been demonstrated using horseradish peroxidase, glucose oxidase^14^ and alkaline phosphatase^15^ with such methods having recently been investigated for biomedical applications^16^ and as a basis for sensing systems^17^. DNA origami can also be used to construct programmable nano-robots that are able to produce a measurable response in the presence of specific (often disease-related) molecular signals. A primary example was demonstrated in 2012 by Douglas *et al.*^18^ whereby a DNA origami nano-robot was used to deliver antibody fragments to cells.

Sharing the same construction material (DNA), aptamer and DNA origami technologies can easily be linked together and aptamers have previously been incorporated into DNA origami^8^,^18^,^19^, although few with clear medical applicability. Nevertheless, aptamers do have significant potential in the development of smart, DNA-origami based medical sensors. These may be devices that utilise aptamers to detect disease-associated molecules and execute a response based on their presence. Aptamers can fulfill this role due to their ability to trigger large scale conformational changes in molecular devices upon specific binding of target molecules as was demonstrated in the nano-robot of Douglas *et al.*^18^ where, crucially, opening to deliver the payload was actuated by DNA aptamer switches. This provides a general blueprint for diagnostic systems that could function in an analogous manner: releasing dyes or other easily detectable molecules in response to binding of disease-related signals to aptamer switches.

We envisage a malaria detecting system consisting of a nanometric DNA origami capsule containing a signaling molecule. The capsule opens and releases the signal only in the presence of a malaria specific molecule (*Pf*LDH, a known, important diagnostic target for malaria^20^,^21^). This is achieved using aptamers for *Pf*LDH that form logic gates integrated into the origami structure. Such a device may be useful as malaria is a major threat to human health and was responsible for over 500,000 deaths in 2013^22^. Resistance to existing drugs including the currently most effective treatments have been reported^23^,^24^ and while progress has been made in vaccine development, a universally applicable vaccine seems a distant prospect at present. The effectiveness of existing and future treatments is improved with early and accurate diagnosis, meaning that the development of increasingly sensitive, robust and sophisticated diagnostic systems is desirable.

As particular aptamers have unique characteristics, each aptamer module incorporated into DNA origami must be assessed to ensure that it maintains its active binding conformation. In the current study we describe the production of a rectangular DNA origami scaffold integrated with the *Pf*LDH-binding DNA aptamer^10^. We demonstrate that upon integration with DNA origami, the aptamers are able to retain the ability to bind specifically to *Pf*LDH. This is the first attempt to use *Pf*LDH in conjunction with DNA origami as an initial step towards the goal of more complex “smart” malaria detection/treatment devices.

## Results and Discussion

### Design of modified DNA origami and aptamer strands

**Figure 1:**
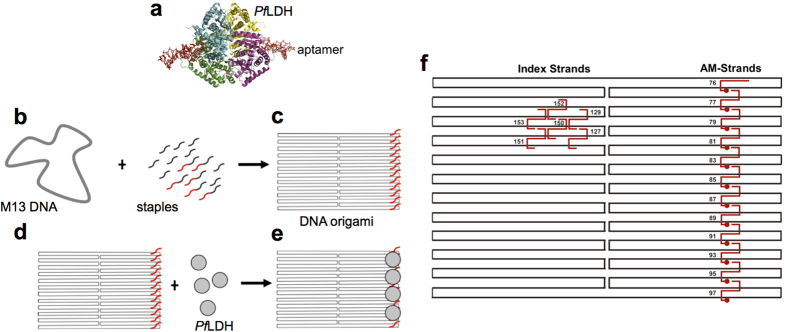
Design and assembly of AM-DNA origami. (**a**) The crystal structure of *Pf*LDH with two aptamers bound (pdb 3ZH2^10^). *Pf*LDH is shown in cartoon format with each monomer coloured a different colour. Aptamer strands are shown in stick format and coloured red. (**b**) DNA origami is made by mixing the M13 DNA strand with staple strands. 12 staple strands were modified with additional sequences (red) corresponding to a linker sequence followed by the aptamer sequence. (**c**) After annealing, the rectangular DNA origami was formed with the 12 aptamer sequences protruding from the surface (red). (**d**) *Pf*LDH (grey circle) is mixed with the modified DNA origami. (**e**) Immobilisation of *Pf*LDH on the rectangular DNA origami surface. (**f**) Schematic of DNA origami strand arrangement. The M13 template is shown in black and the staple strands are shown in red. Red staple strands on the right of the structure are those which were modified by addition of the aptamer sequences while strands 127, 129, 150, 151, 152, 153 are the index staples. Staple strands are named as in Table 1.

We based our DNA origami template on a standard rectangle design^25^ (see supplementary materials for sequences) and employed aptamer sequences known to bind to *Pf*LDH, whose structure and interaction with target protein are known in unprecedented detail^10^. In order to incorporate these aptamers into DNA origami, a staple sequence complementary to part of the M13 DNA origami template strand was added at the 5′ end of the aptamer. We produced 12 different modified aptamers containing 12 staple sequences complementary to M13 DNA at positions that formed a linear pattern along one edge of the DNA rectangle (Fig. 1, Table 1). Previously, a strong position-dependent hybridisation effect was ascribed to electrostatic repulsion between the target and the underlying tile; as well as weaker steric hindrance at the edge of the tile and stronger repulsion between probes in the middle of the tile^25^. By locating aptamers at the edge of the DNA origami we expect to increase protein accessibility. To further increase flexibility of the aptamer, a linker sequence, consisting of 20 thymidines was included between the staple and aptamer sequences.

When the aptamer-modified (AM) DNA origami is on a mica surface it is possible that the aptamer sequences may lie on the surface of the DNA origami facing the mica and hence not visible during atomic force microscopy (AFM) analysis. Although Voigt *et al.* reported that 90–95% of chemically modified DNA origamis had modifications that were facing the solution^26^, for some origami structures we included an index sequence to provide orientation. The index consisted of six aptamers located on the left side of the DNA origami as previously described by Ke *et al.*^25^ (see Table 1, Fig. 1f).

### Aptamer strands attached to DNA origami staples retain *Pf*LDH-binding specificity

**Figure 2:**
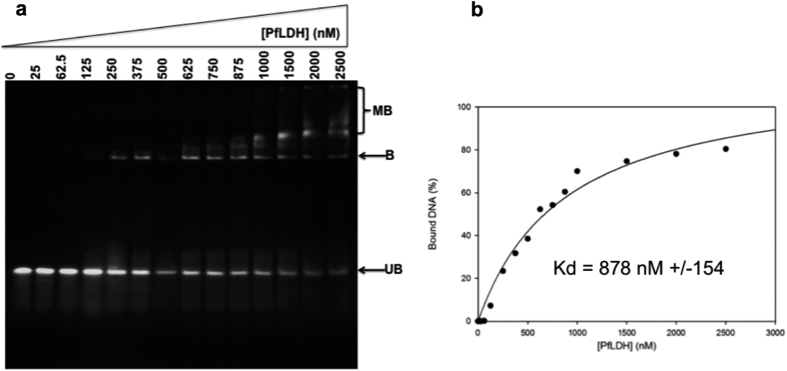
EMSA of aptamer binding to *Pf*LDH: (**a**) Example of an EMSA for aptamer strand 87 (25 nM) binding to *Pf*LDH (0–2500 nM calculated as tetrameric concentration). UB: unbound DNA; B: Bound DNA; MB: multiply bound DNA. (**b**) Binding curve for the gel in **(a**).

For eventual application as part of a diagnostic system for malaria, AM-origami should bind *Pf*LDH but not the human homolog (hLDH). In order to test if the modified aptamers were able to retain binding specificity for *Pf*LDH, we carried out electrophoretic mobility shift assays (EMSA) in the presence of increasing concentrations of either *Pf*LDH or hLDH (see Supplementary Figs S1–S13 (one example of which is shown in Fig. 2) and Table 2). The results show that all strands retain the ability to bind to *Pf*LDH with typical apparent Kds (fitted with a simple 1:1 ligand binding equation) of several hundred nM per subunit. These values are higher than the previously reported Kd of ∼60 nM for unmodified aptamers^10^, although it must be noted that the earlier results were carried out under different experimental conditions. The presence of non-aptamer sequences attached to the aptamer strands is likely responsible for the observed decrease in affinity of the aptamer for its target. Non-interacting sequences at the termini of aptamers have previously been shown to result in a decreased affinity for their targets, likely due to additional unwanted interactions^27^. Significantly, none of the modified aptamers tested were able to bind to hLDH (Supplementary Fig. S13), in agreement with previous results^10^.

Addition of the aptamer sequence to staple strands at different positions on the origami was also investigated (see Supplementary Fig. S14, Supplementary Table S2). These sequences also showed an ability to bind to *Pf*LDH (Supplementary Fig. S15, Table S2) with similar affinities, except for strand 87 which showed weak binding (Supplementary Fig. S15a, Table S2) and strand 121 which showed no binding (Supplementary Fig. S15e, Table S2). Both of these sequences consisted of a central rather than terminal aptamer sequence, flanked at both 5′ and 3′ ends by staple sequences. It is likely that these flanking sequences inhibited the specific interactions required for recognition of the binding site by the aptamer.

### *Pf*LDH-binding AM-staples are able to integrate into a DNA origami structure

**Figure 3:**
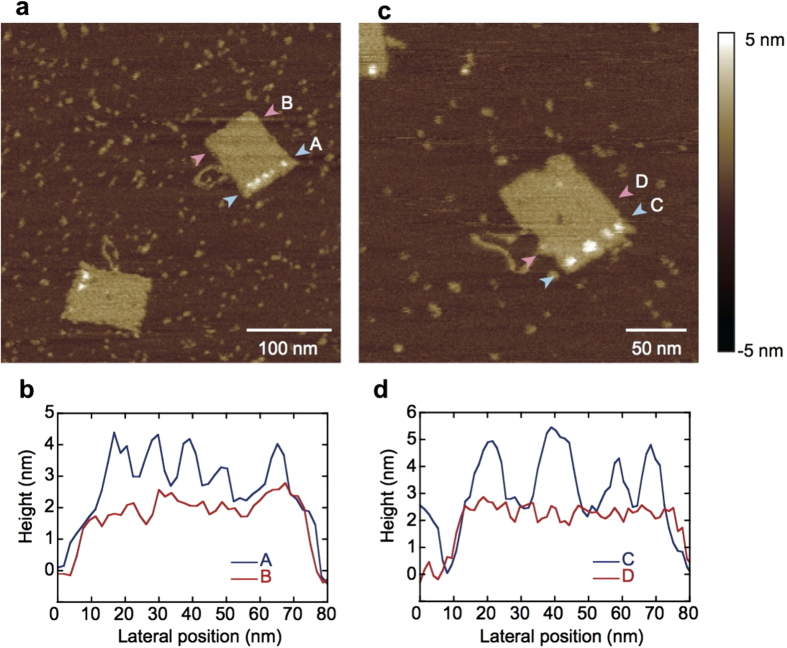
AFM images of AM-DNA origami rectangles in the absence of protein. (**a**) Two DNA origami rectangles are visible with a column of discrete raised areas corresponding to the position of aptamers. Paths used for height profile measurements lie between the pink (“B”) and blue (“A”) arrowheads. (**b**) Height profile plot taken from the height profiles marked in (**a**). Peaks corresponding to the position of the aptamer-modified strands are clearly visible. (**c)** A higher magnification image analyzed in the same way as (**a)** and showing another DNA origami rectangle with four clear aptamer peaks. (**d**) Height profile plot taken from the height profiles marked in (**c)**.

Next, we tested if the AM-staple strands could integrate into the rectangular DNA origami structure. The modified strands were added to the rectangular DNA origami mix, which was annealed according to a standard protocol^28^. The resulting structures were observed using AFM (Fig. 3). Comparison of the DNA origamis with and without AM-staple strands show similar rectangle shapes but where AM-strands were included, a linear area of raised height (approx. +2 nm) is visible near one edge of the rectangle at the expected position, confirming the presence of the active aptamer strands. All DNA origamis had at least one discernible aptamer, however, the maximum number of height peaks varied considerably and was often less than 12 (see also Supplementary Fig. S16). These could represent the total number of aptamers (i.e. some AM-staples did not integrate into the origami structure), or may be due to aptamer-aptamer interaction, resulting in two aptamers appearing as a single structure in AFM. This is quite feasible given their partially complimentary sequences and the fact that they are located on adjacent double strands and hence separated by only approximately 5 nm.

### *Pf*LDH binds specifically at predefined locations to AM-DNA origami

**Figure 4:**
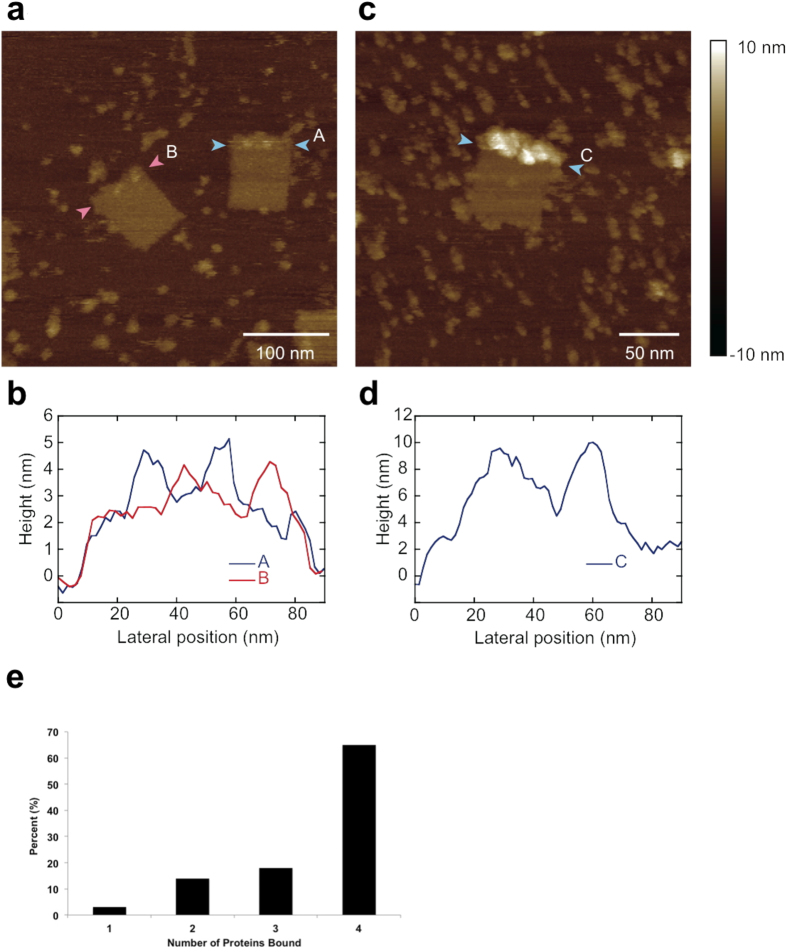
AFM images of AM-DNA origami in the presence of hLDH or *Pf*LDH. (**a)** Two modified DNA origamis in the presence of hLDH. Paths used for height profile measurements lie between the pink (“B”) and blue (“A”) arrowheads. (**b)** Height profile plot taken from the height profiles marked in (**a**). Each origami appears to have two distinct height maxima corresponding to positions of aptamers. (**c)** A modified DNA origami in the presence of *Pf*LDH. Paths used for height profile measurements lie between the blue (“C”) arrowheads. (**d**) Height profile plot taken from the height profiles shown in (c) show a large increase in height due to bound protein. (**e**) Analysis of *Pf*LDH binding to AM-DNA origami showing the percentage of measured AM-DNA origamis containing the indicated number of bound *Pf*LDH enzymes after incubation with an excess of enzyme (n = 100, mean = 3.5, std. dev. = 0.9).

AFM imaging of AM-DNA origami after mixing with hLDH showed no binding of the protein (Fig. 4a,b). In contrast, *Pf*LDH bound specifically to the areas of the DNA origami rectangle corresponding to the location of aptamers, and was visible as a ~5 nm increase in height (Fig. 4c,d). Other areas of the DNA origami rarely showed any evidence of protein binding, even with protein concentrations as high as 1000 nM, presumably due to electrostatic repulsion between the protein (pI 7.12) and the phosphate backbone of the DNA. Most DNA origamis were successfully decorated with one or more proteins: In a sample of 100 AM-origamis in the presence of excess *Pf*LDH no examples lacking bound protein were observed (Fig. 4e). This apparent high yield for protein-bound AM-origami may be expected based on Kd calculations from EMSA experiments. Over repeated observations of large numbers of samples we did observe occasional instances of AM-origami with no protein bound, as may be expected thermodynamically and which could also be due to a minority of origamis that may lie with their modified surfaces facing the mica surface.

Analysis of AM-origamis with *Pf*LDH bound using static AFM (Fig. 4e) showed little evidence for binding of more than four proteins, suggesting that the number of proteins that can bind is limited by protein diameter and/or that each protein may bind simultaneously to two aptamers. This is consistent with the fact the *Pf*LDH tetramer is approximately 9 nm in diameter while the 12 equally spaced aptamers are arranged across the ~60 nm width of the origami rectangle. Some variability in the apparent diameter of proteins is evident and this may be due to occasional binding of proteins as monomers to single aptamers, and, indeed under high speed AFM we were able to observe dissociation of the *Pf*LDH tetramer into monomers (Supplementary Movie File M1).

To test the limits of detection, we carried out the experiments with lower concentrations of *Pf*LDH (750 nM and 500 nM, Supplementary Figs S17 and S18 respectively). Results indicated that binding could still be observed at protein concentration as low as 500 nM (See Supplementary Fig. S18). By comparison, *in vivo* concentrations have been reported as spanning the range 0 ng/mL –22,387.2 ng/mL (mean ± standard deviation 3,917.5 ± 6,120.9 ng/mL) in patient samples^29^. The relatively high lower limit we report in this work is consistent with EMSA findings (Table 2, Fig. 2) and may also be due to the presence of excess staple strands. We attempted to remove these before addition of protein via a range of membrane purification techniques. However, these resulted in production of origami structures devoid of aptamers, presumably due to an unfavourable reaction of the protruding aptamer with the membrane surface.

We also tested an alternative DNA origami-*Pf*LDH binding procedure. In this case, protein was bound to the aptamer prior to integration into the DNA origami structure, a method that allows for protein-aptamer binding in conditions that may not be compatible with DNA origami formation and allows interchangeable aptamer modules to be swiftly incorporated with a universal, premade DNA origami “chassis”. In this two-step assembly process, the first step comprised of incubating the 12 modified aptamer strands with *Pf*LDH, while annealing the remaining staples with the M13mp18 ssDNA backbone as previously. In the second step, the incubated aptamer strand-protein assembly was mixed with the partially folded DNA origami and re-annealed (from 37 °C) to allow the DNA origami structures to self-assemble fully. AFM analysis showed that binding of *Pf*LDH onto the DNA origami surface was successfully achieved using this stepwise addition reaction with results comparable to the one step process (Supplementary Fig. S19).

### Centrifugation removes unbound protein and promotes protein-mediated intra-origami interaction

**Figure 5:**
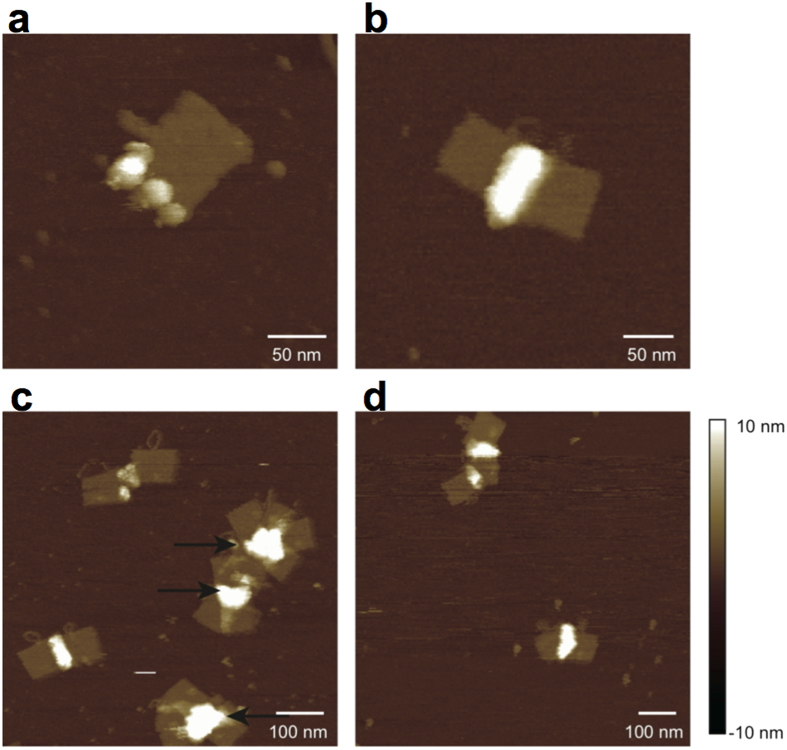
Effects of centrifugation and blood plasma. (**a**–**c**) Centrifugation of modified origamis constructed using 12 aptamers in the presence of *Pf*LDH resulted in a significant decrease in the amount of unbound protein while aptamer-bound protein remained. Centrifugation also promoted aggregation of origami structures, apparently via protein-protein interactions resulting in (**b**) dimerisation or (**c**) larger aggregates (indicated by arrows). (**d**) Presence of blood plasma has little effect on AM-DNA origami integrity or protein binding ability. Linear arrangements of *Pf*LDH are clearly visible and some aggregation occurs due to protein-protein interaction as a result of centrifugation.

To decrease the presence of background noise in AFM images due to unbound protein, we carried out an ultracentrifugation step (see Experimental Procedures). The results (Fig. 5a–c) showed that this was successful in removing the majority of unbound protein from the sample. It also resulted in greater aggregation of individual origami rectangles, apparently mediated by protein-protein interactions between bound proteins.

In order to probe stoichiometry of binding we produced DNA origamis with 4 rather than the maximum 12 aptamers. Modified aptamers 76, 81, 89 and 97 (Fig. 1f) were retained while the remaining modified aptamers were replaced with non-modified counterparts. *Pf*LDH-bound origamis were prepared using the centrifugation step as described above. The results showed that many origamis aggregated, making clear counting difficult in the majority of cases. However, the analysable origamis showed fewer proteins bound compared to the 12-aptamer version, typically no more than two (Supplementary Fig. S20). This could be due to each *Pf*LDH protein binding to two aptamers, one for each binding site. The separation between aptamer binding positions in the case of the 4-aptamer origami is 15–20 nm, with the linker separating the beginning of the aptamer sequence from the origami surface being 20 bases, while the separation between aptamer binding sites within a single *Pf*LDH (as measured between K106 residues of monomers A and C^10^) is approximately 9 nm, which is also the approximate overall diameter for the protein.

### High-Speed AFM reveals dynamics of *Pf*LDH binding to modified DNA origami

**Figure 6:**
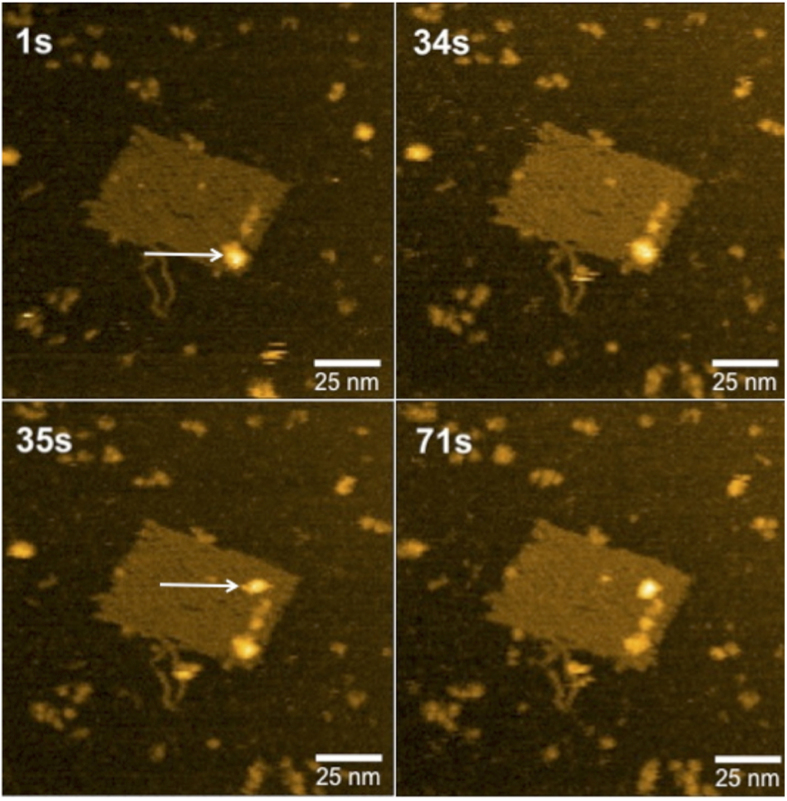
HS-AFM shows dynamics of interaction between AM-origami and *Pf*LDH. Four frames from HS-AFM analysis of AM-origami in the presence of *Pf*LDH (see Supplementary Movie File M5) are shown with time points indicated in seconds. At 1 s, a single AM-origami is visible with one *Pf*LDH (indicated by white arrow) attached to the aptamer-modified region. At 35 s a second *Pf*LDH (indicated by white arrow) binds to the region and both proteins remain in place beyond 71 s (see Supplementary Movie File M5).

In order to gain insight into the dynamics of *Pf*LDH binding to AM-DNA origami we carried out high-speed AFM experiments. We were able to view *Pf*LDH-bound modified DNA origami, without protein bound (Supplementary Movie Files M2, M3) and with protein bound (Supplementary Movie Files M4,M5-M6) including instances where binding/unbinding of protein is visible (Fig. 6, Supplementary Movie Files M5-M6). Overall, bound samples under these conditions appeared stable, without rapid on/off rates.

### DNA aptamers are able to bind *Pf*LDH in the presence of blood plasma

Aptamer-operated DNA origami diagnostics for medical use may have to function in the presence of biological fluids. Therefore we tested the ability of aptamers incorporated into DNA origami to bind to *Pf*LDH in the presence of human blood plasma. Although the densely packed double helical nature of the DNA origami is expected to be resistant to digestion by nucleases^30^, the aptamers protrude from the origami surface and so could potentially be more vulnerable. Plasma was added to solutions containing AM-DNA origami and *Pf*LDH and analysed using AFM (Fig. 5d). The presence of plasma had little effect on the integrity of the DNA origami, in agreement with previous studies showing that DNA origami is stable in cell lysate^31^ and is viable in living animals^32^,^33^. We also found that the presence of plasma had no effect on the ability of the aptamers integrated into DNA origami to bind to *Pf*LDH, consistent with previous reports for the original aptamer alone^11^.

### *Pf*LDH bound to DNA origami retains enzymatic activity

**Figure 7:**
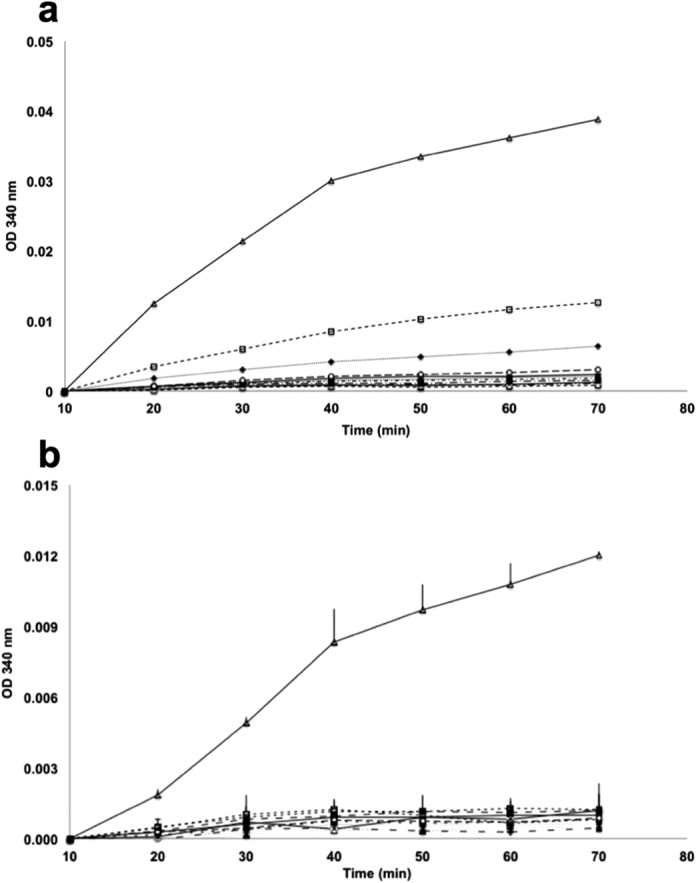
Enzymatic activity of post-ultracentrifugation fractions of AM-DNA origami incubated with *Pf*LDH and without (**a**) or with (**b**) the addition of human blood plasma. Fraction number refers to distance migrated during centrifugation with fraction 19 being less dense than fraction 20 and so on. In both **a** and **b** symbols are as follows: ♦ Fraction 19, ∇ Fraction 20, ▲ Fraction 21, × Fraction 22, ■ Fraction 23, • Fraction 24, + Fraction 25, − Fraction 26, ○ Fraction 27, ♦ Fraction 28, □ Fraction 29, △ Fraction 30.

*Pf*LDH has a well-characterised enzyme activity that can easily be detected. The ability to localise and concentrate this activity to specific, defined regions of programmable nanodevices and structures could be useful in nanometric detectors. DNA aptamers specific to *Pf*LDH have previously been shown to offer the ability to detect the protein in blood samples when coupled with a colorimetric assay^11^. To test if *Pf*LDH similarly retained activity after binding to AM-DNA origami, we separated bound sample from unbound protein using ultracentrifugation. We collected fractions at different depths from the centrifuged sample and tested for lactate dehydrogenase activity. The results (Fig. 7a) showed that activity was highest in the fraction that sedimented most (fraction 30). The same fraction tested in AFM prior to enzymatic assay showed protein bound to AM-DNA origamis with little evidence of free protein (Fig. 5d). Comparison to other fractions with a lower *Pf*LDH activity showed a similar result but with significantly fewer complexes present (Supplementary Fig. S21). Analogous enzyme activity experiments but with the addition of human blood plasma showed similar results (Fig. 7b) where, for fraction 30, enzyme activity was retained at a 2–3 fold lower level compared to absence of plasma (Fig. 7a).

## Conclusions

We have produced a new DNA origami that incorporates a well-defined aptamer able to specifically bind to *Pf*LDH, whose structure is known with Angstrom level precision. This is the first attempt to incorporate a malaria-detecting molecule onto a DNA origami scaffold.

The resulting AM-DNA origami is stable and *Pf*LDH binds specifically at aptamer positions with the stoichiometry of binding limited by the size of the protein complex, with a likelihood of two aptamers binding per protein complex. The resulting structures are stable under a range of conditions including in the presence of human blood plasma. Under high-speed AFM conditions, stable protein-AM-origami binding is observed and protein monomers, which may also be capable of aptamer-binding, are readily formed. The bound proteins retain enzymatic activity, consistent with the crystal structure^10^ which shows that the aptamer binding does not block the substrate binding site of *Pf*LDH.

A well-characterised aptamer specific to *Pf*LDH could be utilised to actuate a sophisticated DNA origami malaria diagnostic device and this work is a first step towards that goal. For example, DNA origami cubes and barrels^18^,^34^ some of which have been shown able to carry cargo are operated by aptamer “keys”^18^. By using *Pf*LDH aptamers in such systems, they could be adapted to carry signal molecules such as fluorescent dyes that are released in the presence of *Pf*LDH when exposed to samples from infected patients. Before such a complex system is constructed, the ability of the aptamer to retain specific *Pf*LDH binding activity when incorporated into an origami system must be demonstrated. In this work we have shown that such specific binding capability is retained when the aptamer is integrated into an origami, providing that it has a flanking staple sequence at only one rather than both termini.

The results of these experiments will allow further development of origami systems actuated by *Pf*LDH-binding aptamers. A DNA origami container is envisaged which will release easily detectable signal molecules from a cavity in the presence of *Pf*LDH.

### Experimental Procedures

#### Materials

M13mp18 single stranded DNA was purchased from Takara Bio Inc. All 226 staple strands and the additional modified 12 aptamer strands were acquired in powder form from Operon (Japan) and diluted to 50 μM with TE buffer and used without further purification. *Pf*LDH and hLDH were produced as described previously^10^.

#### DNA origami assembly

A rectangular-shaped DNA origami structure (~90 × 60 nm) was assembled from a 7.2 kilobase viral M13mp18 single-stranded DNA and 226 staple strands in a buffer containing 1× TAE/Mg^2+^ (40 mM Tris, 20 mM acetic acid, 2 mM EDTA, 12.5 mM magnesium acetate, pH 8). Details of the sequence designs of the staple strands are included in the Supplementary Information (Table S1). The concentrations used for the study were 12 nM of viral genome scaffold and a fivefold molar excess of staples. The relevant non-modified staple strands were replaced with modified counterparts (either AM or index strands) at the same concentration.

DNA mixtures were annealed using a PCR machine (BioRad DNA Engine) at the following settings: for the one step assembly and 1^st^ annealing: 90 °C for 10 min, with a subsequent temperature decrease of 1 °C/min until 25 °C; for the two step assembly and 2^nd^ annealing (re-annealing): 37 °C for 10 min followed by a decrease of 1 °C/min until 25 °C, repeated five times.

#### Electrophoretic mobility shift assay

The aptamer strand-protein binding assay was performed by incubating a fixed concentration of aptamer staple (25 nM) with varying concentrations of *Pf*LDH protein (0 nM–2500 nM) in 25 mM Tris-HCl containing 100 mM NaCl, 20 mM imidazole at pH 7.5 and allowed to incubate at 25 °C for 1 h. Samples were resolved using a 12% non-denaturing polyacrylamide (37.5:1) gel with a 5% stacking gel layer. Samples were loaded for analysis with 20 ng of aptamer in each lane and run at 80 V, 8 °C for 4 h. The gels were subsequently stained with SYBR Gold for 25 min and visualised on a UVP Benchtop (2UV Transilluminator) at 302 nm. Gels were analyzed by ImageJ^35^ by measuring the change in the intensity of the unbound DNA band. Data was subsequently fitted using a 1:1 ligand binding equation equivalent in form to the Michaelis-Menten equation.

#### Origami-*Pf*LDH complexes

AM-DNA origami (12 nM) was mixed with *Pf*LDH protein (1000 nM) in 25 mM Tris-HCl containing 100 mM NaCl, 20 mM imidazole at pH 7.5 and allowed to incubate at 25 °C for 1 h. The resulting DNA origami-*Pf*LDH complexes had a final concentration of ~8.6 nM.

#### Origami-plasma complexes

Human plasma (Sigma Aldrich) was reconstituted in 1 ml of deionised water. Aliquots ranging from 1 μl to 40 μl were incubated with both DNA origami alone and DNA origami – protein complexes to make up a total of 100 μl of sample, at 25 °C for 1 h. The plasma containing DNA origami samples were further purified by rate-zonal centrifugation.

#### Purification by rate-zonal centrifugation

DNA origami-protein complexes were purified from excess staples, unbound protein and human plasma using a glycerol gradient through rate-zonal centrifugation according to the method reported previously^36^. Quasi-continuous glycerol gradients between 45–15% were prepared in 5 ml ultracentrifuge tubes, by overlaying seven layers of glycerol solution (45% at the bottom, 700 μl per layer, 5% concentration decrease per layer) and left overnight at 4 °C. Samples (100 μl each) containing 10% glycerol were loaded onto the gradients and centrifuged (Beckman Optima Max ultracentrifuge) at 50000 rpm for 2 h at 4 °C via a swinging –bucket rotor (MLS-50). Subsequently, 30 fractions equal in volume were collected and visualised under AFM, with aliquots being used for the enzymatic assay.

#### Enzymatic assay

*Pf*LDH-catalysed conversion of lactate to pyruvate was spectrophotometrically determined by following the reduction of NAD^+^ at 340 nm. 90 μl of assay mixture containing 2 mM NAD^**+**^ (Sigma-Aldrich) and 71 mM sodium lactate (Sigma-Aldrich) in phosphate buffered saline (PBS) was incubated with an aliquot of the post rate-zonal centrifugation fractions (10 μl) in a 96-well plate at 25 °C. The change in absorbance was recorded every 10 min using a microplate reader (SpectraMax M2, Molecular Devices, CA, USA).

#### AFM imaging

DNA structures were imaged by AFM. Subsequent to annealing and incubation, an aliquot (1–2 μl) of sample was deposited onto the surface of a freshly cleaved mica with 9.9 mm diameter (Agar Scientific, UK) and left to adsorb for 5 min, followed by the addition of 50 μl of HEPES/Mg^2+^ buffer (40 mM HEPES, 10 mM NiCl_2_, 12.5 mM magnesium acetate, pH 7.6). The AFM used was a Multimode-AFM with NanoScope5 controller (Bruker Corp., CA, USA). Samples were imaged using a micro cantilever with spring constant ~0.1 N/m (BL-AC40TS-C, Olympus, Japan) at a scan rate of 1.95 Hz in peak force tapping mode in fluid.

#### High speed AFM

Additional imaging was performed by High Speed AFM (HS-AFM) at Kanazawa University. The tips used (BL-AC10DS or BL-AC7DS-KU2, Olympus, Japan) were made of amorphous carbon and were each sharpened prior to use through a combination of plasma etching and electron-beam deposition. Imaging was performed as described previously^37^.

#### Analysis of formed DNA origamis

For DNA origami including aptamers but not protein, AFM results were examined which allowed the number of protruding aptamers, discernible as discrete regions with a raised height profile, to be counted. For DNA origami structures containing aptamer and *Pf*LDH, 1000 nM *Pf*LDH protein was incubated with 12 nM DNA origami at room temperature for 1 h, and subsequently visualised under AFM. One hundred DNA origami tiles were chosen at random from the existing AFM images and the number of proteins immobilised on each tile, discernible as discrete regions with a raised height profile, were evaluated visually. One, two and three proteins were countable as discrete structures. Given that the diameter of a single *Pf*LDH allows a maximum of four proteins to lie across the width of the DNA origami, protein structures on origami that were visible only as a continuous region of raised height were classified as four proteins.

## Additional Information

**How to cite this article**: Godonoga, M. *et al.* A DNA aptamer recognising a malaria protein biomarker can function as part of a DNA origami assembly. *Sci. Rep.* **6**, 21266; doi: 10.1038/srep21266 (2016).

## Supplementary Material

Supplementary Information

Supplementary Movie M1

Supplementary Movie M2

Supplementary Movie M3

Supplementary Movie M4

Supplementary Movie M5

Supplementary Movie M6

## Figures and Tables

**Table 1:** Sequences of the 12 aptamer staple strands and the 6 index staple strands.

Aptamer ID	Sequence
76	TATCACCGTACTCAGG AGGTTTAGCGGGGTTT TTTT TTTT TTTT TTTT TTTT *CTGGGCGGTAGAACCATAGTGACCCAG CCGTCTAC*
77	TGCTCAGTCAGTCTCT GAATTTACCAGGAGGT TTTT TTTT TTTT TTTT TTTT *CTGGGCGGTAGAACCATAGTGACCCAG CCGTCTAC*
79	TGAGGCAGGCGTCAGA CTGTAGCGTAGCAAGG TTTT TTTT TTTT TTTT TTTT *CTGGGCGGTAGAACCATAGTGACCCAG CCGTCTAC*
81	CCGGAAACACACCACG GAATAAGTAAGACTCC TTTT TTTT TTTT TTTT TTTT *CTGGGCGGTAGAACCATAGTGACCCAG CCGTCTAC*
83	TTATTACGGTCAGAGG GTAATTGAATAGCAGC TTTT TTTT TTTT TTTT TTTT *CTGGGCGGTAGAACCATAGTGACCCAG CCGTCTAC*
85	CTTTACAGTTAGCGAA CCTCCCGACGTAGGAA TTTT TTTT TTTT TTTT TTTT *CTGGGCGGTAGAACCATAGTGACCCAG CCGTCTAC*
87	TCATTACCCGACAATA AACAACATATTTAGGC TTTT TTTT TTTT TTTT TTTT *CTGGGCGGTAGAACCATAGTGACCCAG CCGTCTAC*
89	AGAGGCATAATTTCAT CTTCTGACTATAACTA TTTT TTTT TTTT TTTT TTTT *CTGGGCGGTAGAACCATAGTGACCCAG CCGTCTAC*
91	TATGTAAACCTTTTTT AATGGAAAAATTACCT TTTT TTTT TTTT TTTT TTTT *CTGGGCGGTAGAACCATAGTGACCCAG CCGTCTAC*
93	GAGCAAAAACTTCTGA ATAATGGAAGAAGGAG TTTT TTTT TTTT TTTT TTTT *CTGGGCGGTAGAACCATAGTGACCCAG CCGTCTAC*
95	CGGAATTATTGAAAGG AATTGAGGTGAAAAAT TTTT TTTT TTTT TTTT TTTT *CTGGGCGGTAGAACCATAGTGACCCAG CCGTCTAC*
97	CTAAAGCAAGATAGAA CCCTTCTGAATCGTCT TTTT TTTT TTTT TTTT TTTT *CTGGGCGGTAGAACCATAGTGACCCAG CCGTCTAC*
127	CCAAATCACTTGCCCT **TCCTCTTTTGAGGAACAAGTTTTCTTGT** GACGAGAACGCCAAAA
129	AAACGAAATGACCCCC **TCCTCTTTTGAGGAACAAGTTTTCTTGT** AGCGATTATTCATTAC
150	ACGAGTAGTGACAAGA **TCCTCTTTTGAGGAACAAGTTTTCTTGT** ACCGGATATACCAAGC
151	AGTAATCTTAAATTGG **TCCTCTTTTGAGGAACAAGTTTTCTTGT** GCTTGAGAGAATACCA
152	GCGAAACATGCCACTA **TCCTCTTTTGAGGAACAAGTTTTCTTGT** CGAAGGCATGCGCCGA
153	ATACGTAAAAGTACAA **TCCTCTTTTGAGGAACAAGTTTTCTTGT** CGGAGATTTCATCAAG

The aptamer staple strands (76, 77, 79, 81, 83, 85, 87, 89, 91, 93, 95, 97) were modified to include the sequence of the aptamer previously reported to bind to *Pf*LDH. Aptamer sequences are shown in italics. The index staple strands are 127, 129, 150, 151, 152, 153. Index modification sequences are shown in bold.

**Table 2:** Apparent Kds of *Pf*LDH binding to each aptamer as determined from EMSA.

Aptamer ID	Kd (nM)
76	965 +/− 265
77	726 +/− 186
79	826 +/− 300
81	970 +/− 254
83	1090 +/−183
85	713 +/− 161
87	878 +/− 154
89	750 +/− 95
91	984 +/− 177
93	647 +/− 128
95	846 +/− 161
97	760 +/− 126
