# Supplementary material for: A DNA aptamer recognising a malaria protein biomarker can function as part of a DNA origami assembly
Source: Sci Rep. 2016 Feb 19;6:21266. doi: 10.1038/srep21266 (PMC4759581; doi:10.1038/srep21266)
Supplement: Supplementary Information [file srep21266-s1.pdf]

A DNA aptamer recognising a malaria protein biomarker can function as part of a DNA origami assembly

Maia Godonoga[1,2], Ting-Yu Lin[1#], Azusa Oshima[3], Koji Sumitomo[3], Marco S. L. Tang[4], Yee-Wai Cheung[4], Andrew B. Kinghorn[4], Roderick M. Dirkzwager[4], Cunshan Zhou[5], Akinori Kuzuya[6], Julian A. Tanner[4] and Jonathan G. Heddle[1,7]*

[1] Heddle Initiative Research Unit, RIKEN, Saitama, 351-0198, Japan

[2] Department of Life Science and Medical Bioscience, Waseda University, 2-2 Wakamatsu-cho, Shinjuku, Tokyo 162-8480, Japan

[3]NTT Basic Research Laboratories, NTT Corporation, 3-1 Morinosato Wakamiya, Atsugi, Kanagawa 243-0198, Japan

[4] School of Biomedical Sciences, Li Ka Shing Faculty of Medicine, The University of Hong Kong, Pokfulam, Hong Kong SAR, China

[5]School of Food and Biological Engineering, Jiangsu University, No.301 Xuefu Road, Zhenjiang 212013, China

[6]Department of Chem. Mater. Eng., Kansai University, 3-3-35 Yamate, Suita, Osaka 564-8680,Japan

[7]Malopolska Centre of Biotechnology, Jagiellonian University, Gronostajowa 7, 30–387, Krakow, Poland

[#]Current address Malopolska Centre of Biotechnology, Jagiellonian University, Gronostajowa 7, 30–387, Krakow, Poland

* To whom correspondence should be addressed, email jonathan.heddle@uj.edu.pl

Supplementary Information

Supplementary Methods

Electrophoretic mobility shift

The electrophoretic mobility shift assay (EMSA) was performed as described in the main text.

Two-Step assembly process

Aptamer-modified DNA origamis were assembled using a two-step assembly process. The first step comprised of incubating the 12 modified aptamer strands with *Pf*LDH protein, while annealing the remaining staples with the M13mp18 ssDNA backbone as previously (90 °C for 10 min, with a subsequent temperature decrease of 1 °C/min until 25 °C). In the second step, the incubated aptamer strand-protein assembly was mixed with the partially folded DNA origami and re-annealed (from 37 °C for 10 min followed by a decrease of 1 °C/minute until 25 °C, repeated five times) to allow the DNA origami structures to self-assemble fully.

DNA origami assembly

The DNA origami assembled in the presence of 12 aptamers was prepared as described in the main text and structures were further visualised using HS- AFM (description in the main text).

Preparation of aptamer-modified DNA origami in the presence of *Pf*LDH

Aptamer-modified DNA origami in the presence of 500 nM and 750 nM *Pf*LDH were prepared by incubating 12 nM of aptamer-modified DNA origami with 500 nM and 750 nM of protein in 25 mM Tris-HCl containing 100 mM NaCl, 20 mM imidazole at pH 7.5, and allowed to incubate at 25 °C for 1 h. Structures were imaged using AFM.

Methods for Supplementary Movie Files

Imaging was performed in 100 mM NaCl and 20 mM imidazole at pH 7.5 on a high speed AFM using oscillation amplitudes around 2 to 6 $nm_{p\text{-}p}$ with continuous

optimization of all parameters (feedback control, scan rate and scan size), in order to enhance the image quality and minimize the tip force effect on the imaged structures. All High-speed AFM images and movies were processed using Igor Pro (Wave Metrics) software developed in the Ando group at Kanazawa University without enhanced image filtering or any additional processing.

Supplementary Movie File M1: 5 µl of *Pf*LDH protein (5.4 µM) was added to the mica surface and visualised using 1x TAE/Mg^{2+} (40 mM Tris, 20 mM acetic acid, 2 mM EDTA, 12.5 mM magnesium acetate, pH 8) buffer. Imaging was performed using HS-AFM.

Supplementary Movie File M2 and Supplementary Movie File M3: A 10x-diluted (1.2 nM) sample of DNA origami modified to contain 12 aptamers was deposited onto freshly cleaved mica and incubated for 5 min. The sample was further washed using HEPES/Mg^{2+} buffer (40 mM HEPES, 10 mM $NiCl_2$, 12.5 mM magnesium acetate, pH 7.6) and imaged under HS-AFM as previously described.

Supplementary Movie File M4: A 0.21 nM (diluted from a 40x concentrated stock) sample of DNA origami – *Pf*LDH complex was left to adsorb onto freshly cleaved mica for 5 min and further washed with HEPES/Mg^{2+} buffer (40 mM HEPES, 10 mM $NiCl_2$, 12.5 mM magnesium acetate, pH 7.6). Imaging was performed under HS-AFM.

Supplementary Movie File M5: A 50x-diluted (0.24 nM) sample of DNA origami modified to include 12 aptamers was deposited onto freshly cleaved mica and left to

adsorb for 5 min, with further addition of HEPES/Mg^{2+} (40 mM HEPES, 10 mM $NiCl_2$, 12.5 mM magnesium acetate, pH 7.6) buffer. During HS-AFM imaging, 3 µl of *Pf*LDH protein (5.4 µM concentration) was added onto the sample and imaging was resumed.

Supplementary Movie File M6: 0.21 nM (diluted from a 40x concentrated stock) of DNA origami – *Pf*LDH complex was left to adsorb onto freshly cleaved mica for 5 min and further washed with HEPES/Mg^{2+} buffer (40 mM HEPES, 10 mM $NiCl_2$, 12.5 mM magnesium acetate, pH 7.6). The sample was further diluted in 25 mM Tris-HCl containing 100 mM NaCl and 20 mM imidazole at pH 7.5. During HS-AFM imaging 10 µl of KCl (3 M) was added to the sample chamber, which led to the dissociation of protein from the DNA origami surface.

Supplementary References

1 Schneider, C. A., Rasband, W. S. & Eliceiri, K. W. NIH Image to ImageJ: 25 years of image analysis. *Nat. Methods* **9**, 671-675 (2012).

2 Ke, Y., Lindsay, S., Chang, Y., Liu, Y. & Yan, H. Self-assembled water-soluble nucleic acid probe tiles for label-free RNA hybridization assays. *Science* **319**, 180-183 (2008).

Supplementary Movie Files Legends

Supplementary Movie File M1: High speed AFM showing *Pf*LDH protein tetramers dissociating into monomers. Movie shown at five frames per second.

Supplementary Movie File M2: High speed AFM of 12-aptamer modified DNA origami. The linearly arranged aptamers are clearly visible. Movie shown at five frames per second.

Supplementary Movie File M3: High speed AFM showing a wide field of view of several 12-aptamer modified DNA origamis. The linearly arranged aptamers are clearly visible. Movie shown at three frames per second.

Supplementary Movie File M4: High speed AFM showing 12-aptamer modified DNA origami with *Pf*LDH proteins bound. The origami shows some deterioration in structure presumable due to the action of the AFM tip. Movie shown at one frame per second.

Supplementary Movie File M5: High speed AFM showing 12-aptamer modified DNA origami with *Pf*LDH proteins bound. Initially a single *Pf*LDH protein is bound. A second protein binds at approx. 35 s. Movie shown at five frames per second.

Supplementary Movie File M6: High speed AFM showing a single *Pf*LDH binding to 12-aptamer modified DNA origami. The presence of KCl in this sample appears to decrease protein interaction with the mica surface but also with the AM-origami. Movie shown at three frames per second.

Supplementary Figure S1

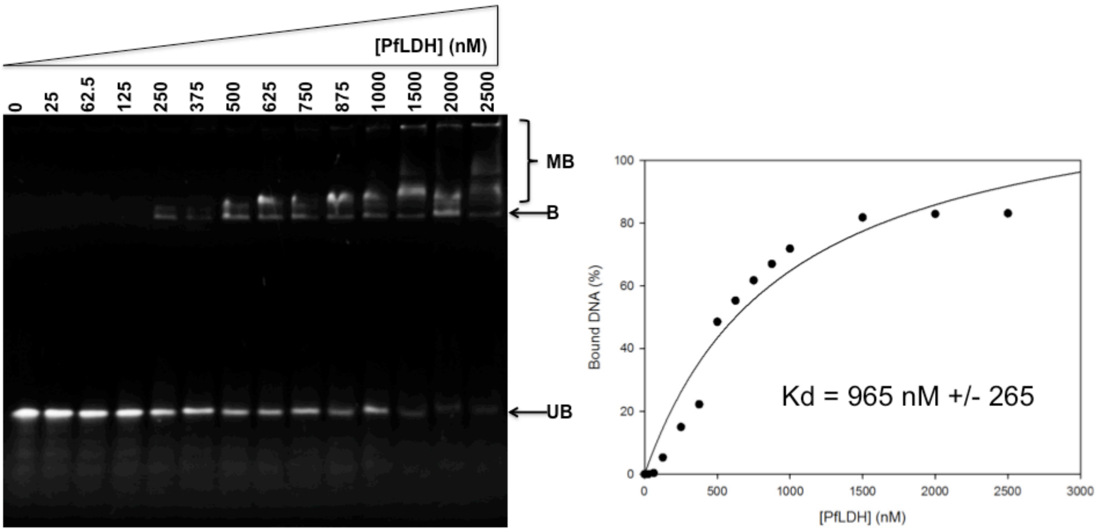

Supplementary Figure S1. Left, EMSA for aptamer strand 76 (25 nM) binding to *Pf*LDH (0-2500 nM calculated as tetrameric concentration). UB – unbound DNA; B – bound DNA; MB – multiply bound DNA. Right, K_d calculation graph.

Supplementary Figure S2

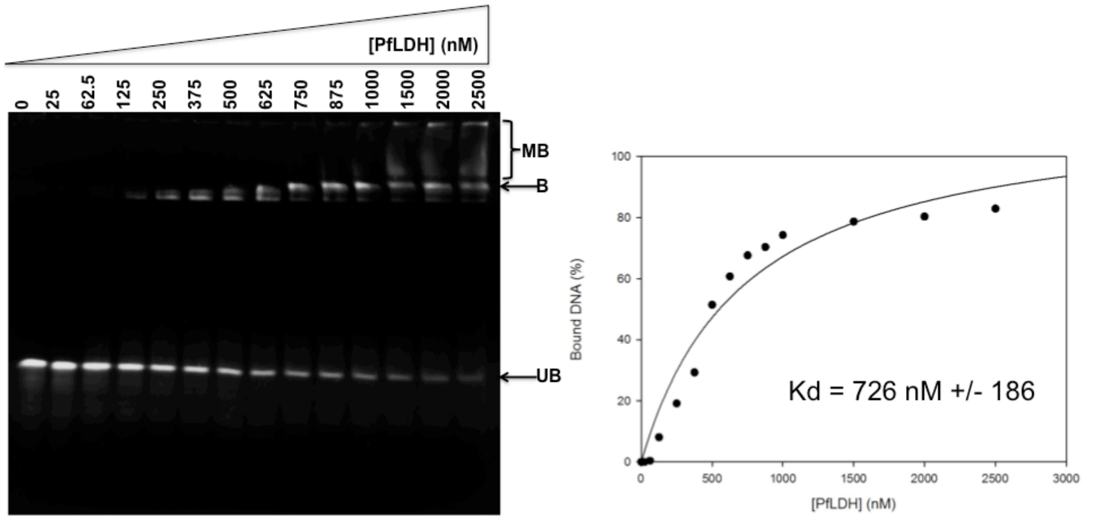

Supplementary Figure S2. Left, EMSA for aptamer strand 77 (25 nM) binding to *Pf*LDH (0-2500 nM calculated as tetrameric concentration). UB – unbound DNA; B – bound DNA; MB – multiply bound DNA. Right, K_d calculation graph.

Supplementary Figure S3

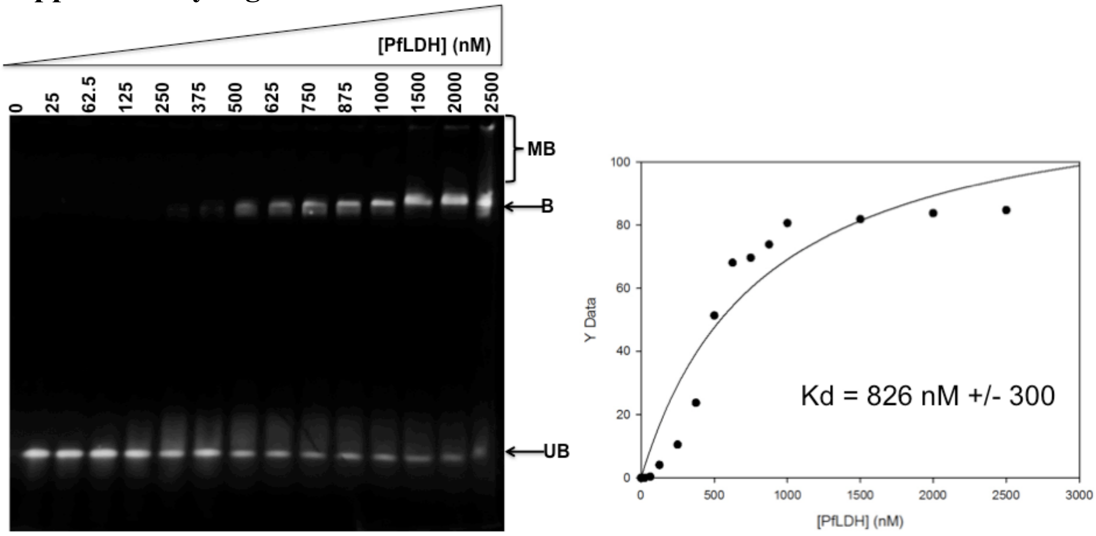

Supplementary Figure S3. Left, EMSA for aptamer strand 79 (25 nM) binding to *Pf*LDH (0-2500 nM calculated as tetrameric concentration). UB – unbound DNA; B – bound DNA; MB – multiply bound DNA. Right, K_d calculation graph.

Supplementary Figure S4

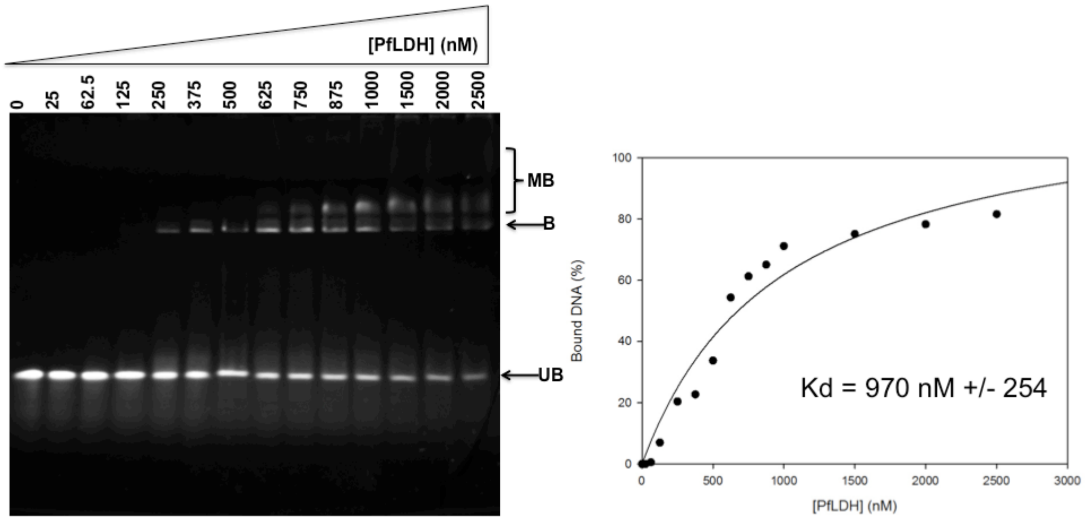

Supplementary Figure S4. Left, EMSA for aptamer strand 81 (25 nM) binding to *Pf*LDH (0-2500 nM calculated as tetrameric concentration). UB – unbound DNA; B – bound DNA; MB – multiply bound DNA. Right, K_d calculation graph

Supplementary Figure S5

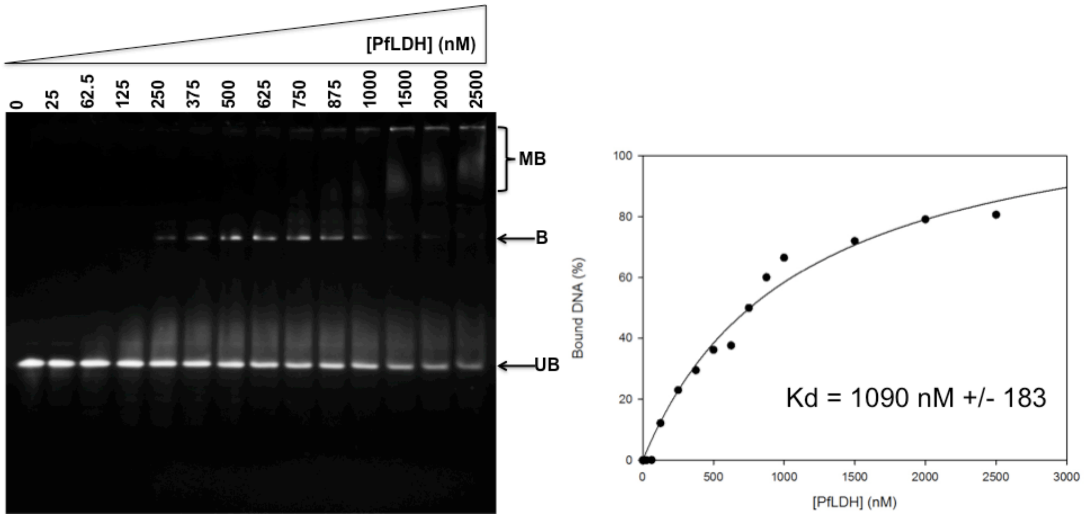

Supplementary Figure S5. Left, EMSA for aptamer strand 83 (25 nM) binding to *Pf*LDH (0-2500 nM calculated as tetrameric concentration). UB – unbound DNA; B – bound DNA; MB – multiply bound DNA. Right, K_d calculation graph

Supplementary Figure S6

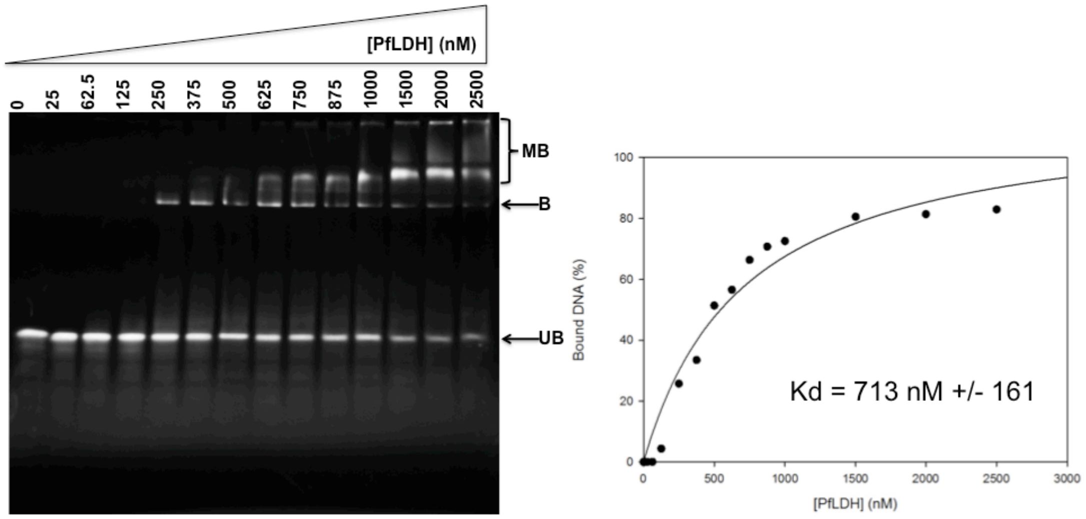

Supplementary Figure S6. Left, EMSA for aptamer strand 85 (25 nM) binding to *Pf*LDH (0-2500 nM calculated as tetrameric concentration). UB – unbound DNA; B – bound DNA; MB – multiply bound DNA. Right, K_d calculation graph

Supplementary Figure S7

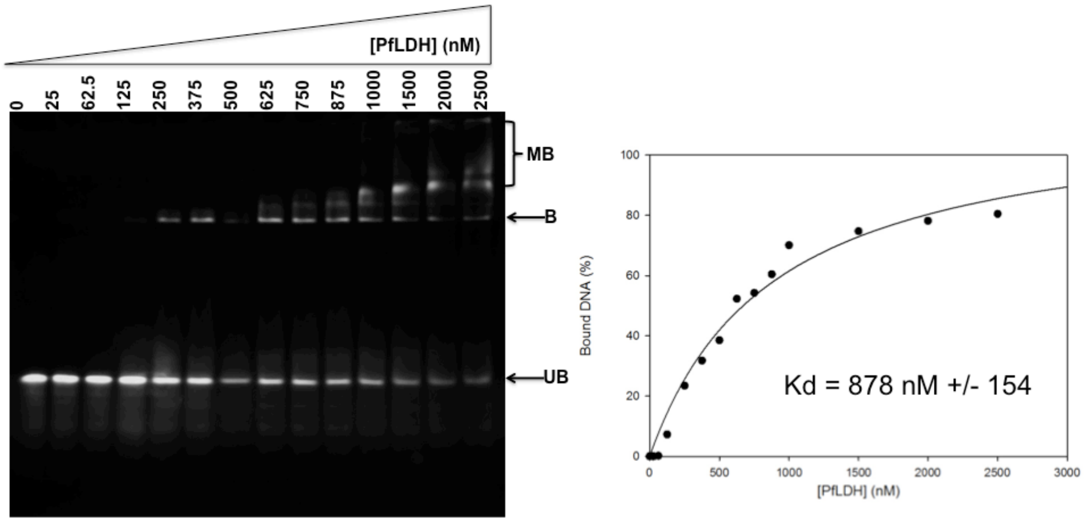

Supplementary Figure S7. Left, EMSA for **a**ptamer strand 87 (25 nM) binding to *Pf*LDH (0-2500 nM calculated as tetrameric concentration). UB – unbound DNA; B – bound DNA; MB – multiply bound DNA. Right, K_d calculation graph. This figure is reproduced as an example result in main text Fig.2

Supplementary Figure S8

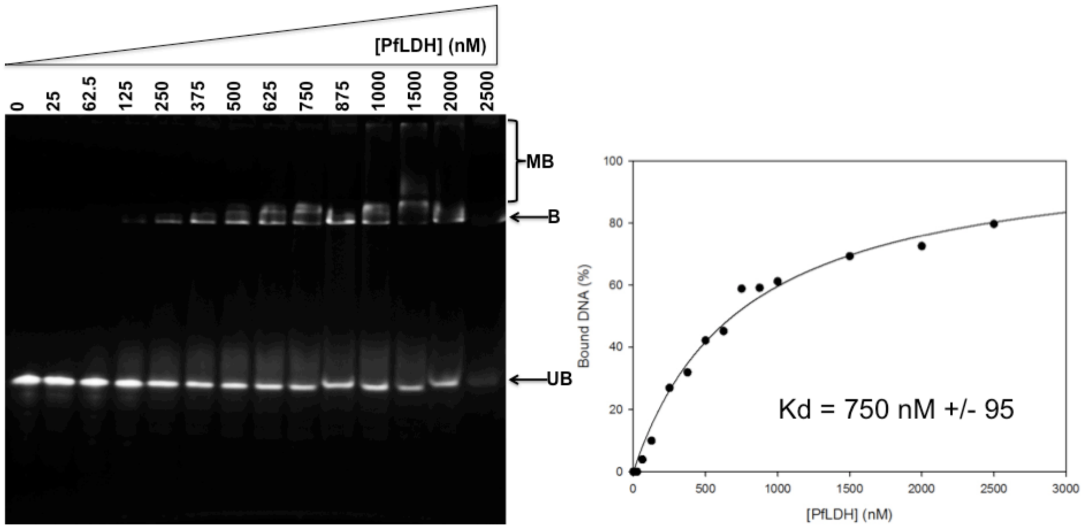

Supplementary Figure S8. Left, EMSA for aptamer strand 89 (25 nM) binding to *Pf*LDH (0-2500 nM calculated as tetrameric concentration). UB – unbound DNA; B – bound DNA; MB – multiply bound DNA. Right, K_d calculation graph

Supplementary Figure S9

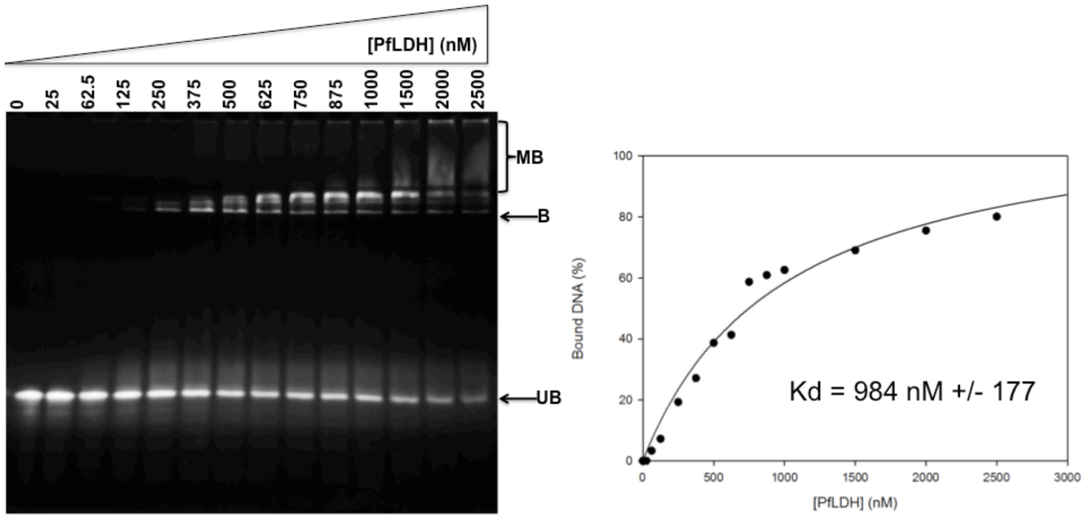

Supplementary Figure S9. Left, EMSA for aptamer strand 91 (25 nM) binding to *Pf*LDH (0-2500 nM calculated as tetrameric concentration). UB – unbound DNA; B – bound DNA; MB – multiply bound DNA. Right, K_d calculation graph

Figure S10

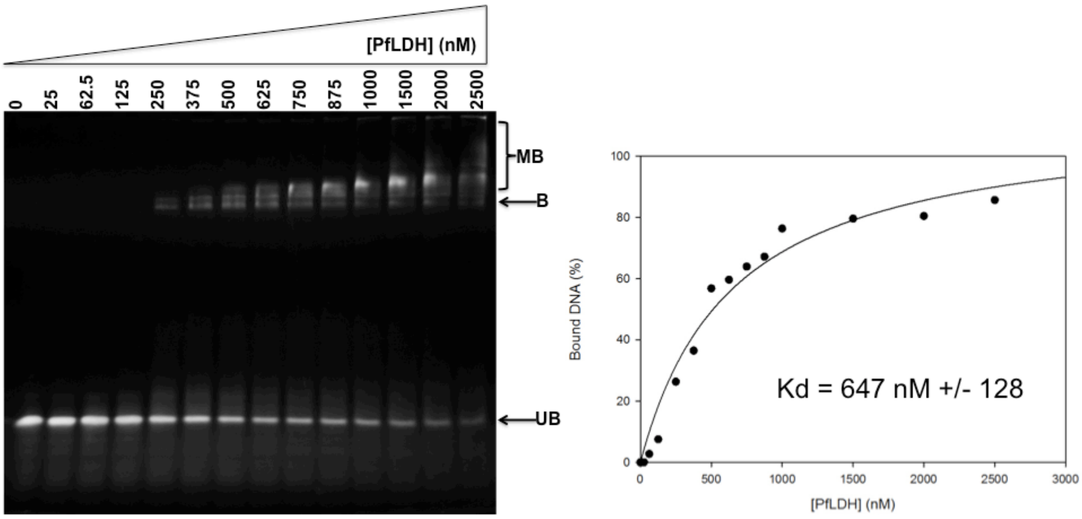

Supplementary Figure S10. Left, EMSA for aptamer strand 93 (25 nM) binding to *Pf*LDH (0-2500 nM calculated as tetrameric concentration). UB – unbound DNA; B – bound DNA; MB – multiply bound DNA. Right, K_d calculation graph

Supplementary Figure S11

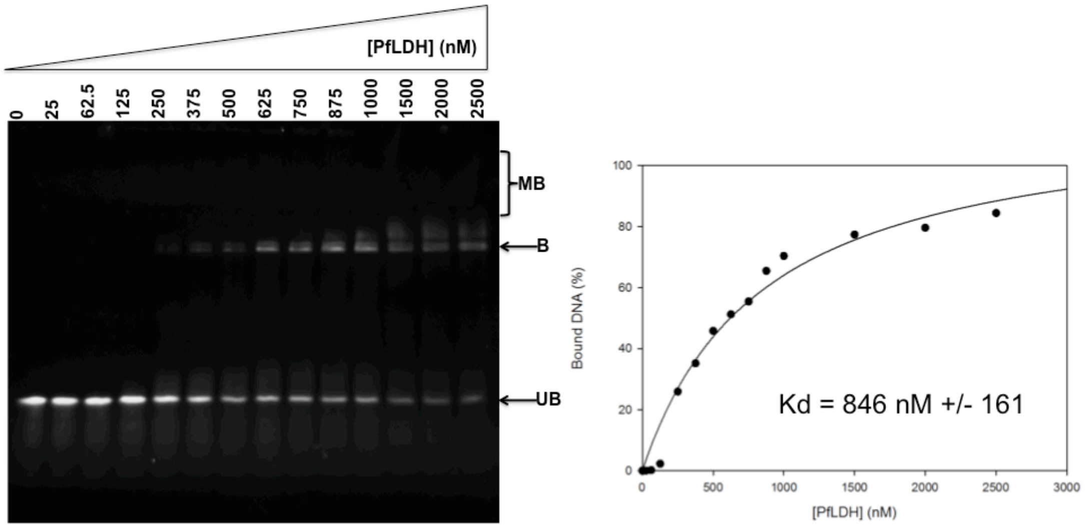

Supplementary Figure S11. Left, EMSA for aptamer strand 95 (25 nM) binding to *Pf*LDH (0-2500 nM calculated as tetrameric concentration). UB – unbound DNA; B – bound DNA; MB – multiply bound DNA. Right, K_d calculation graph

Supplementary Figure S12

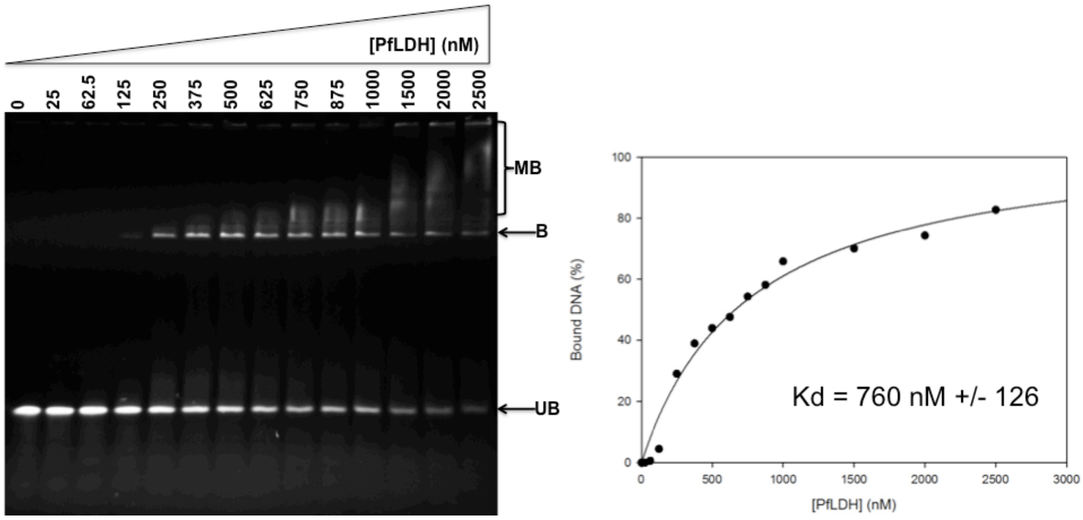

Supplementary Figure S12. Left, EMSA for aptamer strand 97 (25 nM) binding to *Pf*LDH (0-2500 nM calculated as tetrameric concentration). UB – unbound DNA; B – bound DNA; MB – multiply bound DNA. Right, K_d calculation graph

Supplementary Figure S13

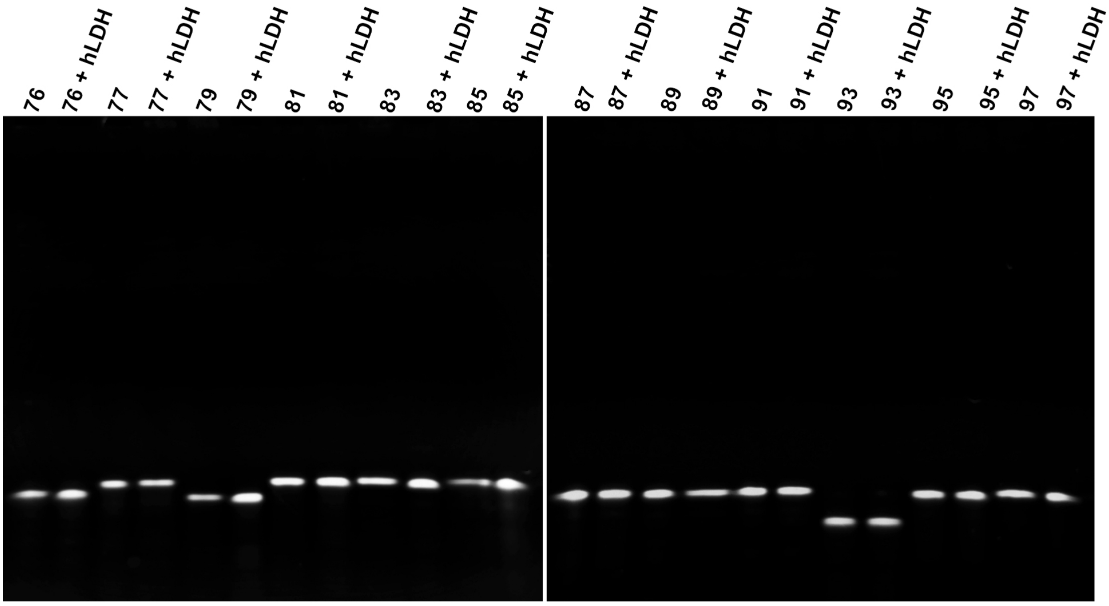

Supplementary Figure S13: Two EMSA gels for all 12 AM-staples (25 nM) with hLDH (1000 nM) showing no binding. Each lane is marked with a number identifying the DNA strand and "hLDH" indicates lanes with the protein present as control.

Supplementary Figure S14

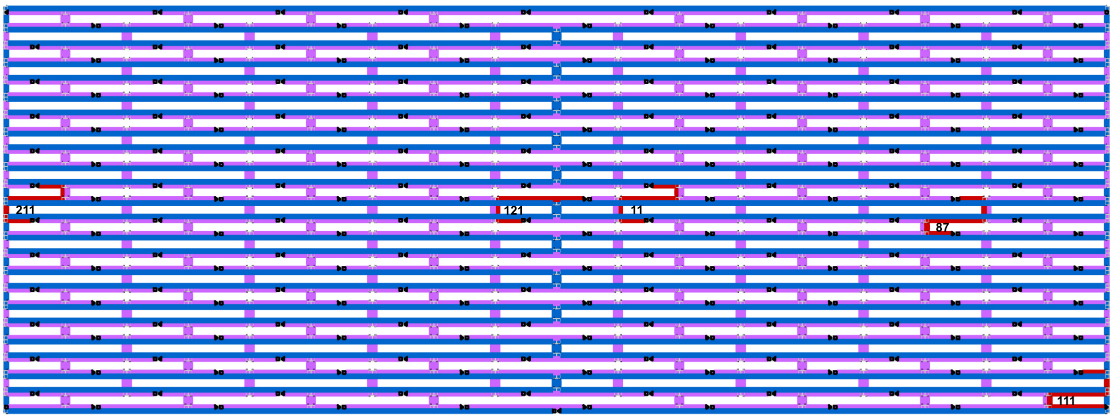

Supplementary Figure S14: Additional modified staple strands. Map of DNA origami showing M13 phage strand in blue and staple strands in violet. Additional staple strands modified with the aptamer sequence are shown in red and are 11, 87, 111, 121, 211.

Supplementary Figure S15

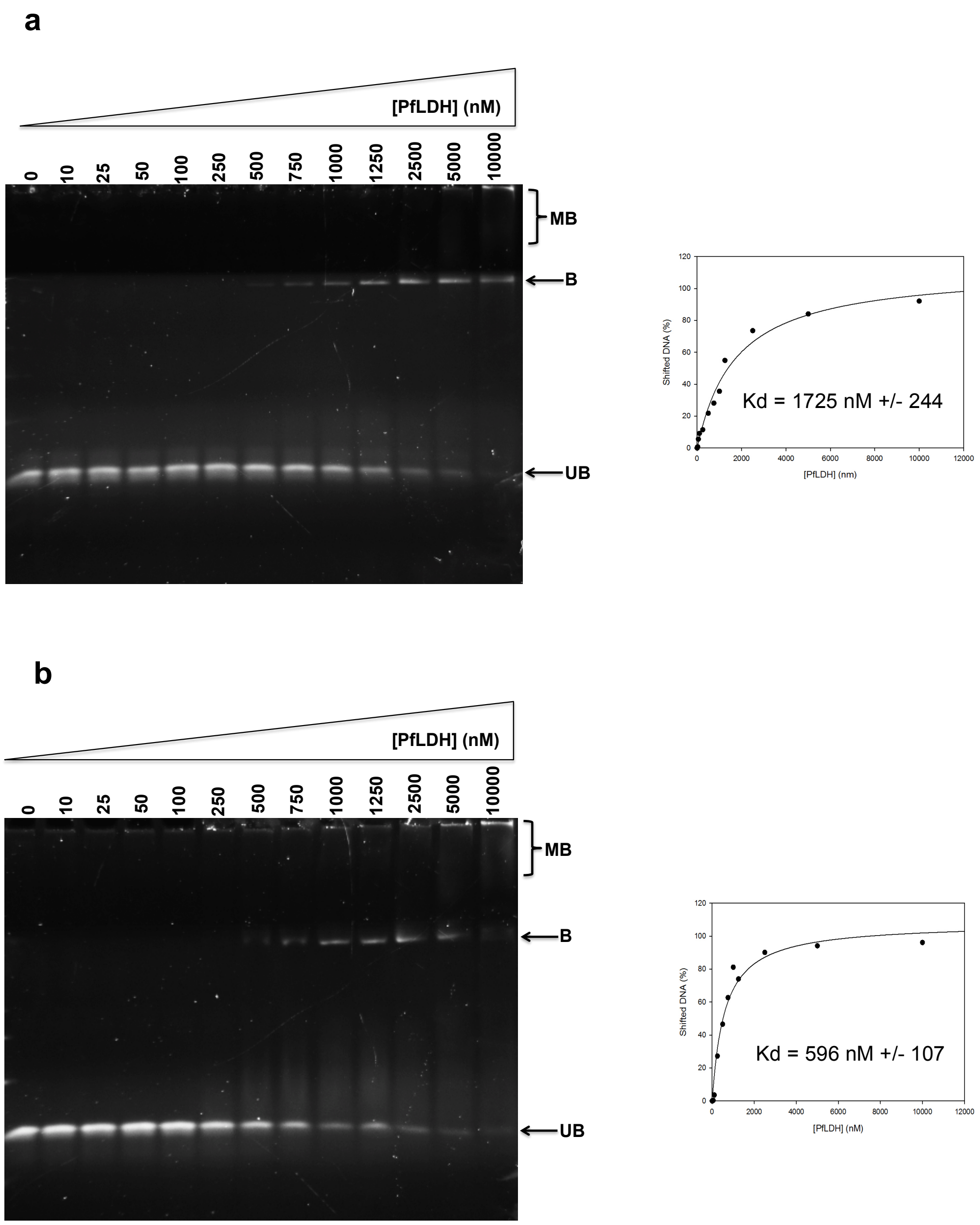

c

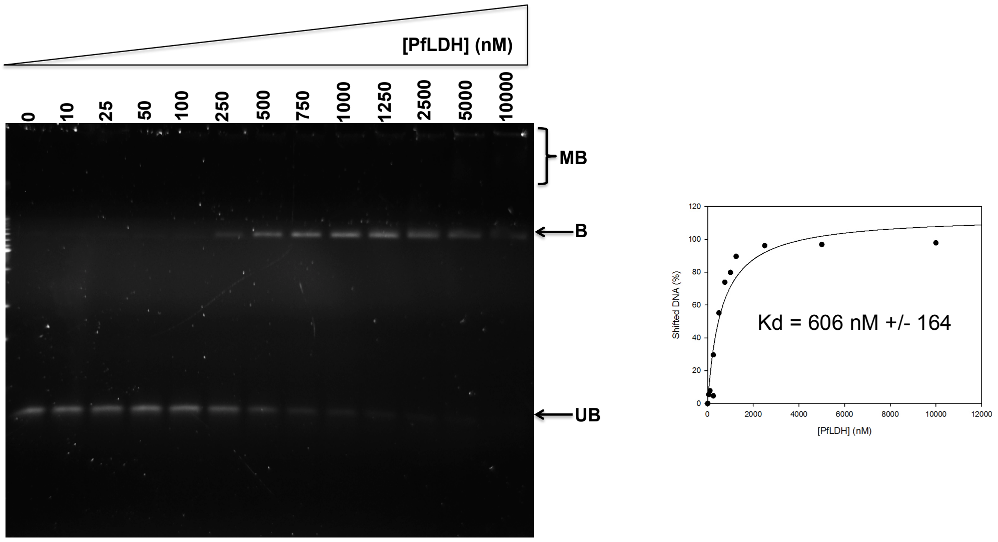
[PfLDH] (nM)
0
10
25
50
100
250
500
750
1000
1250
2500
5000
10000
MB
B
UB
Shifted DNA (%)
[PfLDH] (nM)
Kd = 606 nM +/- 164

d

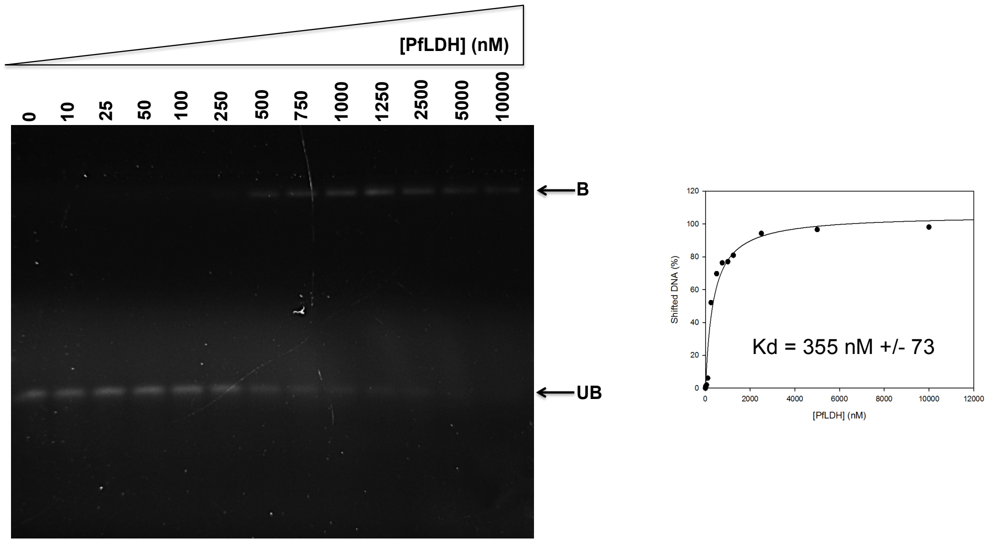
[PfLDH] (nM)
0
10
25
50
100
250
500
750
1000
1250
2500
5000
10000
B
UB
Shifted DNA (%)
[PfLDH] (nM)
Kd = 355 nM +/- 73

e

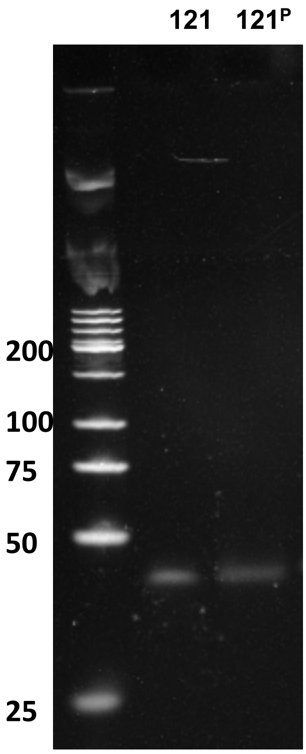

Supplementary Figure S15: Titration of *Pf*LDH against various aptamer-modified staple strands at 4 °C. **a.** Strand 87**, b.** Strand 11, **c.** Strand 111**, d.** Strand 211. Graphs accompanying each gel are a quantification of the % of DNA shifted, fitted with 1:1 ligand binding curve. Lanes in Gel **a** are labeled with [*Pf*LDH] (nM). The same labels apply to gels **b**-**d**. **e.** Demonstration of lack of *Pf*LDH binding by strand 121: Staple strand 121 is indicated by number and the presence of the protein by a superscript “P”. 2.5 µM *Pf*LDH was used and the concentration of aptamer-modified staple strand was kept constant at 25 nM. The first lane is a low molecular weight DNA ladder, sizes of selected bands (in base pairs) are marked.

Supplementary Figure S16

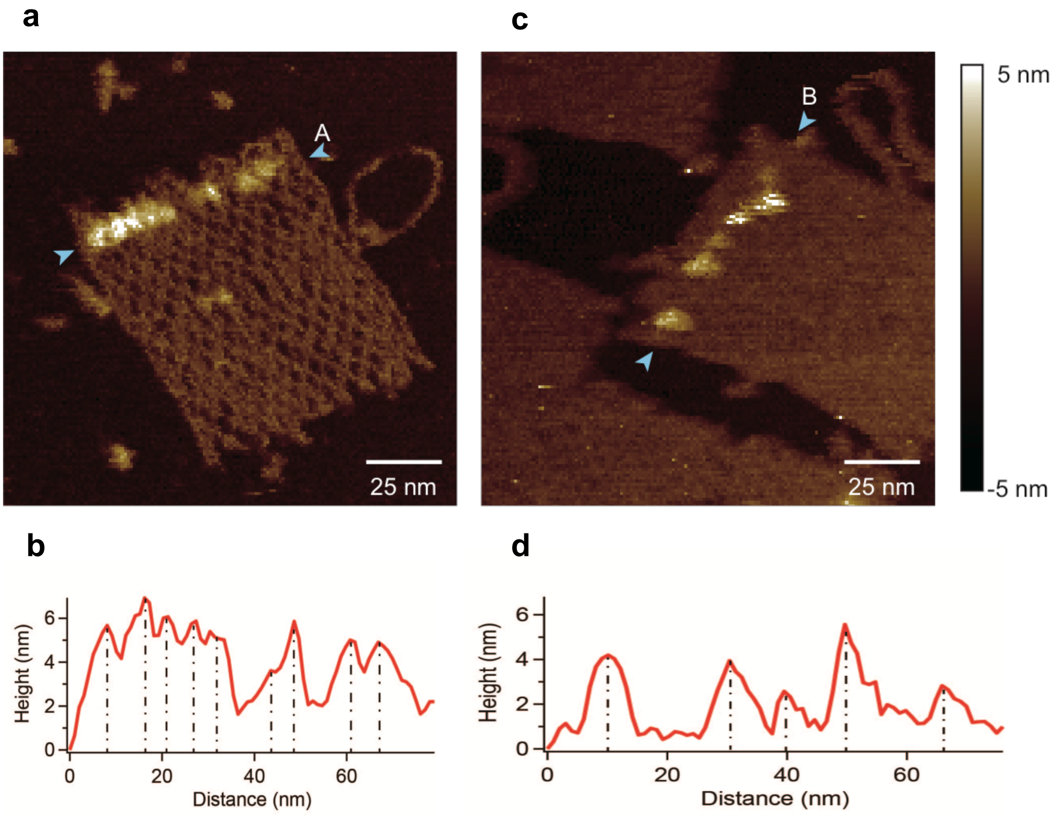

Supplementary Figure S16: Variable numbers of aptamers are visible on DNA origami: **a** and **c** show two different AFM images of two different DNA origami rectangles, both assembled in the presence of 12 aptamers. Arrowheads show the termini of lines used for height profile measurements. **b** and **d** show height profile plots taken from the height profiles shown in **a** and **c** respectively. Peaks are marked with dotted lines. Nine identifiable peaks corresponding to nine aptamers are discernible in **b** and were marked using dotted lines. Five identifiable peaks were marked using dotted lines in **d**.

Supplementary Figure S17

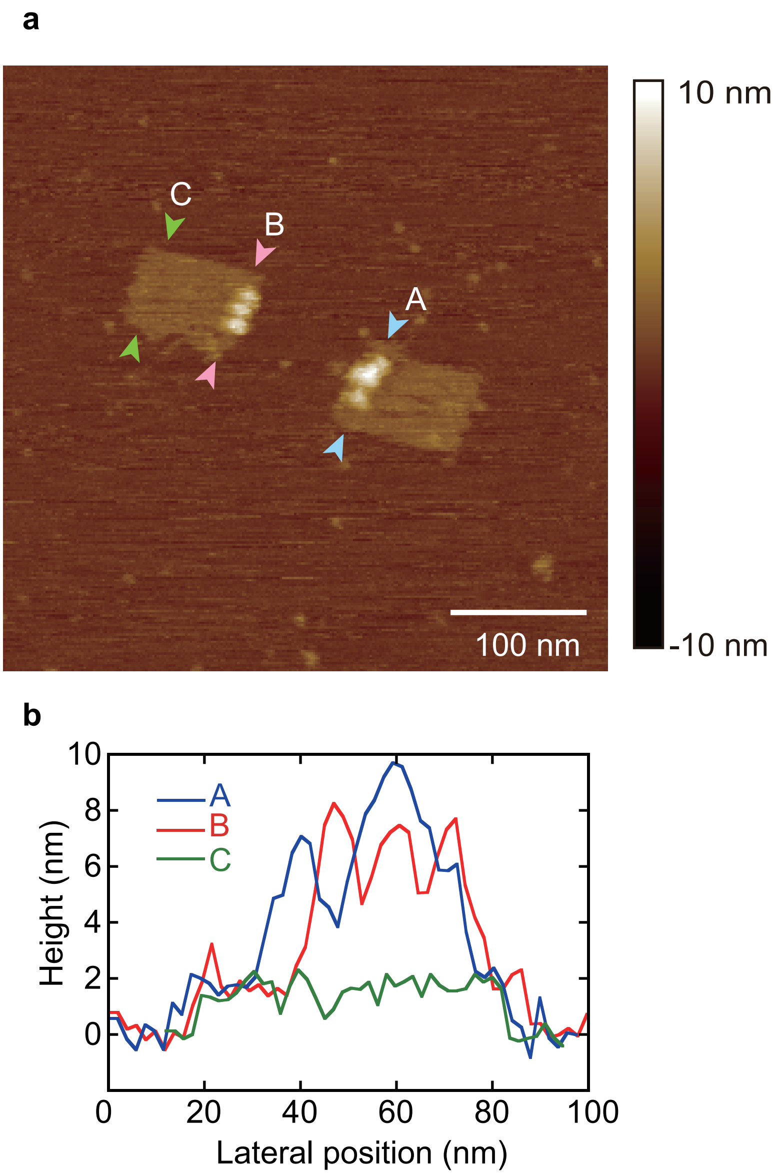

Supplementary Figure S17. a. shows an AFM image of the modified DNA origami in the presence of 750 nM *Pf*LDH with coloured arrowheads showing the termini of the paths used for height profile measurements. **b**. Height profile plot taken from the height profiles shown in **a**. The colour of each profile line corresponds to the colours marked on the line in **a**.

Supplementary Figure S18

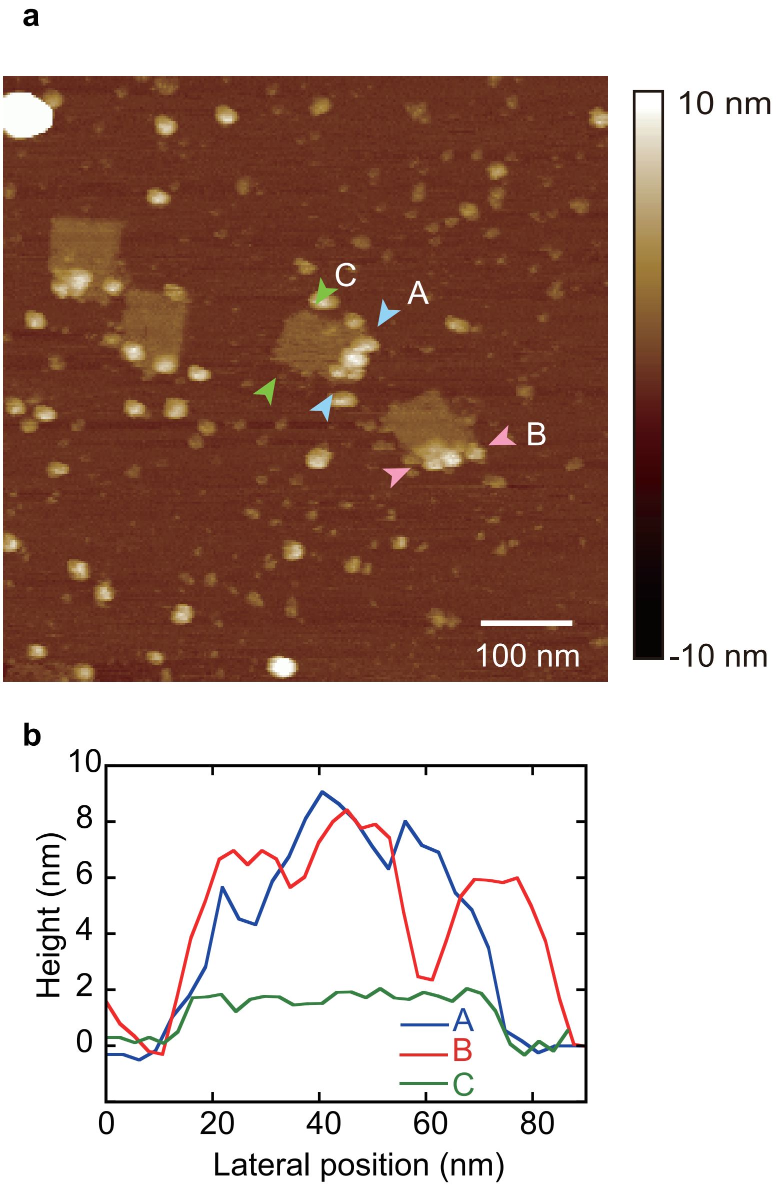

Supplementary Figure S18. a shows an AFM image of the modified DNA origami in the presence of 500 nM *Pf*LDH with coloured arrowheads showing the termini of the paths used for height profile measurements. **b**. Height profile plot taken from the height profiles shown in **a**. The colour of each profile line corresponds to the colours marked on the line in **b**.

Supplementary Figure S19

a

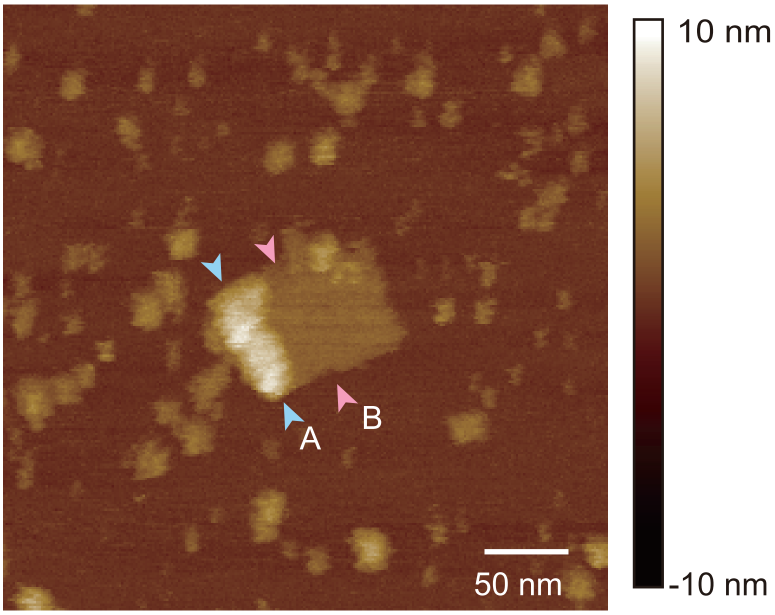

b

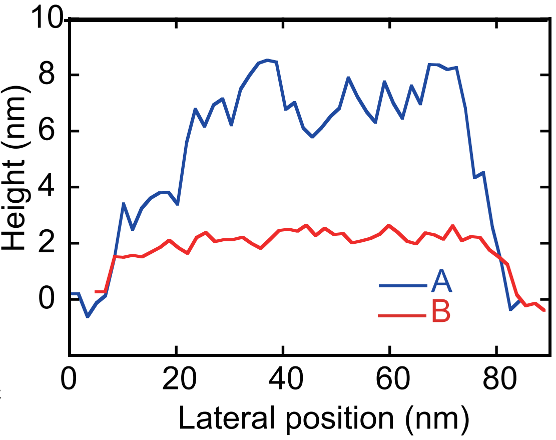

c

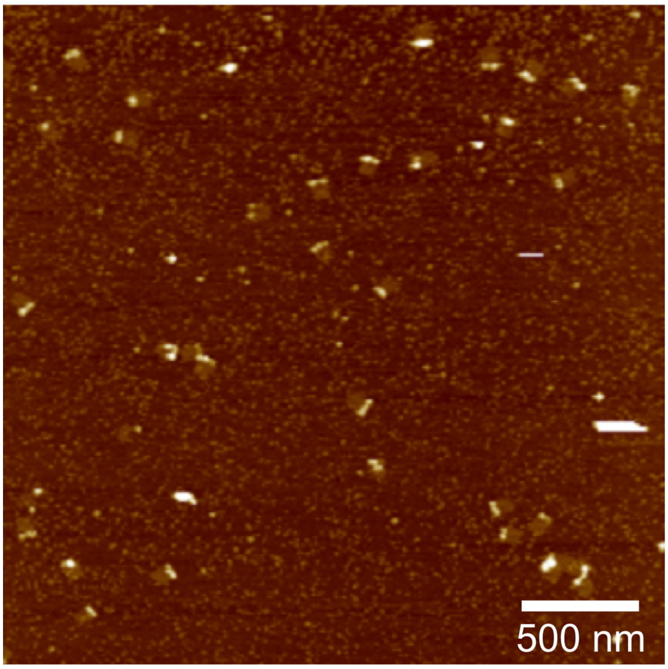

Supplementary Figure S19: AFM images of an aptamer-modified DNA origami incubated in the presence of *Pf*LDH, assembled using two-step assembly and not subject to a centrifugation step. **a**. A DNA origami rectangle is visible with a column of discrete raised areas corresponding to the position of aptamers bound to protein. Blue and pink arrowheads mark the termini of the paths used for height measurements. **b**. Height profile plot taken from the height profiles shown in **a. c**. A lower magnification AFM image of AM-DNA origami in the presence of *Pf*LDH showing a large number of DNA origamis with protein bound in the characteristic pattern.

Supplementary Figure S20

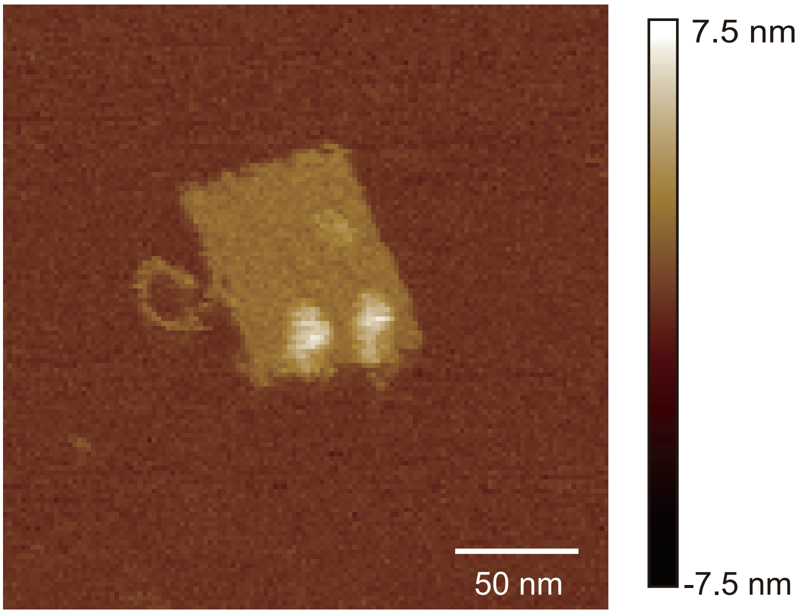

Supplementary Figure S20: Aptamer-modified DNA origami containing 4 aptamers and index after mixing with *Pf*LDH and purification by ultracentrifugation. The results clearly show two *Pf*LDH proteins bound at the expected aptamer positions.

Supplementary Figure S21

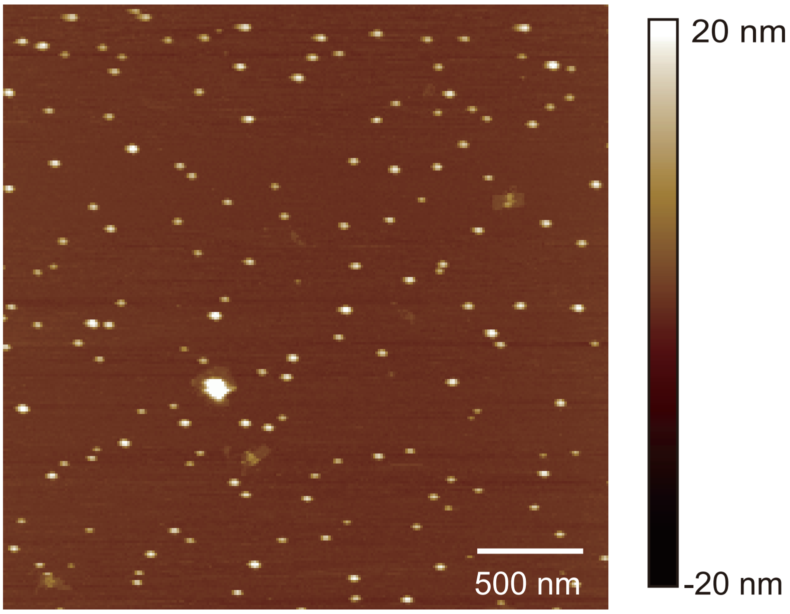

Supplementary Figure S21: AFM image of aptamer-modified DNA origami constructed in the presence of 12 aptamers after centrifugation. In this case, a less dense fraction (fraction 20) was imaged: occasional DNA origami structures (rectangles) and a large amount of unbound protein (white dots) were observed.

Supplementary Table S1

Sequence of the staple strands used for the assembly of the rectangular DNA origami were identical to those previously described[2] and are as follows:

1	CAAGCCCAATAGGAAC CCATGTACAAACAGTT
2	AATGCCCCGTAACAGT GCCCGTATCTCCCTCA
3	TGCCTTGACTGCCTAT TTCGGAACAGGGATAG
4	GAGCCGCCCCACCACC GGAACCGCGACGGAAA
5	AACCAGAGACCCTCAG AACCGCCAGGGGTCAG
6	TTATTCATAGGGAAGG TAAATATT CATTCAGT
7	CATAACCCGAGGCATA GTAAGAGC TTTTTAAG
8	ATTGAGGGTAAAGGTG AATTATCAATCACCGG
9	AAAAGTAATATCTTAC CGAAGCCCTTCCAGAG
10	GCAATAGCGCAGATAG CCGAACAATTCAACCG
11	CCTAATTTACGCTAAC GAGCGTCTAATCAATA
12	TCTTACCAGCCAGTTA CAAAATAAATGAAATA
13	ATCGGCTGCGAGCATG TAGAAACCTATCATAT
14	CTAATTTATCTTTCCT TATCATTCATCCTGAA
15	GCGTTATAGAAAAAGC CTGTTTAG AAGGCCGG
16	GCTCATTTTCGCATTA AATTTTTG AGCTTAGA
17	AATTACTACAAATTCT TACCAGTAATCCCATC
18	TTAAGACGTTGAAAAC ATAGCGATAACAGTAC
19	TAGAATCCCTGAGAAG AGTCAATAGGAATCAT
20	CTTTTACACAGATGAA TATACAGTAAACAATT
21	TTTAACGTTCGGGAGA AACAATAATTTTCCCT
22	CGACAACTAAGTATTA GACTTTACAATACCGA
23	GGATTTAGCGTATTAA ATCCTTTGTTTTCAGG
24	ACGAACCAAAACATCG CCATTAAA TGGTGGTT
25	GAACGTGGCGAGAAAG GAAGGGAA CAAACTAT
26	TAGCCCTACCAGCAGA AGATAAAAACATTTGA
27	CGGCCTTGCTGGTAAT ATCCAGAACGAACTGA
28	CTCAGAGCCACCACCC TCATTTTCCTATTATT
29	CTGAAACAGGTAATAA GTTTTAACCCCTCAGA

30 AGTGTACTTGAAAGTA TTAAGAGGCCGCCACC

31 GCCACCACTCTTTTCA TAATCAAACCGTCACC

32 GTTTGCCACCTCAGAG CCGCCACCGATACAGG

33 GACTTGAGAGACAAAA GGGCGACAAGTTACCA

34 AGCGCCAACCATTTGG GAATTAGATTATTAGC

35 GAAGGAAAATAAGAGC AAGAAACAACAGCCAT

36 GCCCAATACCGAGGAA ACGCAATAGGTTTACC

37 ATTATTTAACCCAGCT ACAATTTTCAAGAACG

38 TATTTTGCTCCCAATC CAAATAAGTGAGTTAA

39 GGTATTAAGAACAAGA AAAATAATTAAAGCCA

40 TAAGTCCTACCAAGTA CCGCACTCTTAGTTGC

41 ACGCTCAAAATAAGAA TAAACACCGTGAATTT

42 AGGCGTTACAGTAGGG CTTAATTGACAATAGA

43 ATCAAAATCGTCGCTA TTAATTAACGGATTCG

44 CTGTAAATCATAGGTC TGAGAGACGATAAATA

45 CCTGATTGAAAGAAAT TGCGTAGACCCGAACG

46 ACAGAAATCTTTGAAT ACCAAGTTCCTTGCTT

47 TTATTAATGCCGTCAA TAGATAATCAGAGGTG

48 AGATTAGATTTAAAAG TTTGAGTACACGTAAA

49 AGGCGGTCATTAGTCT TTAATGCGCAATATTA

50 GAATGGCTAGTATTAA CACCGCCTCAACTAAT

51 CCGCCAGCCATTGCAA CAGGAAAAATATTTTT

52 CCCTCAGAACCGCCAC CCTCAGAACTGAGACT

53 CCTCAAGAATACATGG CTTTTGATAGAACCAC

54 TAAGCGTCGAAGGATT AGGATTAGTACCGCCA

55 CACCAGAGTTCGGTCA TAGCCCCCGCCAGCAA

56 TCGGCATTCCGCCGCC AGCATTGACGTTCCAG

57 AATCACCAAATAGAAA ATTCATATATAACGGA

58 TCACAATCGTAGCACC ATTACCATCGTTTTCA

59 ATACCCAAGATAACCC ACAAGAATAAACGATT

60 ATCAGAGAAAGAACTG GCATGATTTTATTTTG

61 TTTTGTTTAAGCCTTA AATCAAGAATCGAGAA

62 AGGTTTTGAACGTCAA AAATGAAAGCGCTAAT

63 CAAGCAAGACGCGCCT GTTTATCAAGAATCGC

64 AATGCAGACCGTTTTT ATTTTCATCTTGCGGG

65 CATATTTAGAAATACC GACCGTGTTACCTTTT

66 AATGGTTTACAACGCC AACATGTAGTTCAGCT

67 TAACCTCCATATGTGA GTGAATAAACAAAATC

68 AAATCAATGGCTTAGG TTGGGTTACTAAATTT

69 GCGCAGAGATATCAAA ATTATTTGACATTATC

70 AACCTACCGCGAATTA TTCATTTCCAGTACAT

71 ATTTTGCGTCTTTAGG AGCACTAAGCAACAGT

72 CTAAAATAGAACAAAG AAACCACCAGGGTTAG

73 GCCACGCTATACGTGG CACAGACAACGCTCAT

74 GCGTAAGAGAGAGCCA GCAGCAAAAAGGTTAT

75 GGAAATACCTACATTT TGACGCTCACCTGAAA

76 TATCACCGTACTCAGG AGGTTTAGCGGGGTTT

77 TGCTCAGTCAGTCTCT GAATTTACCAGGAGGT

78 GGAAAGCGACCAGGCG GATAAGTGAATAGGTG

79 TGAGGCAGGCGTCAGA CTGTAGCGTAGCAAGG

80 TGCCTTTAGTCAGACG ATTGGCCTGCCAGAAT

81 CCGGAAACACACCACG GAATAAGTAAGACTCC

82 ACGCAAAGGTCACCAA TGAAACCAATCAAGTT

83 TTATTACGGTCAGAGG GTAATTGAATAGCAGC

84 TGAACAAACAGTATGT TAGCAAACTAAAAGAA

85 CTTTACAGTTAGCGAA CCTCCCGACGTAGGAA

86 GAGGCGTTAGAGAATA ACATAAAAGAACACCC

87 TCATTACCCGACAATA AACAACATATTTAGGC

88 CCAGACGAGCGCCCAATAGCAAGCAAGAACGC

89 AGAGGCATAATTTCAT CTTCTGACTATAACTA

90 TTTTAGTTTTTCGAGC CAGTAATAAATTCTGT

91 TATGTAAACCTTTTTT AATGGAAAAATTACCT

92 TTGAATTATGCTGATG CAAATCCACAAATATA

93 GAGCAAAAACTTCTGA ATAATGGAAGAAGGAG

94 TGGATTATGAAGATGA TGAAACAAAATTTCAT

95 CGGAATTATTGAAAGG AATTGAGGTGAAAAAT

96 ATCAACAGTCATCATA TTCCTGATTGATTGTT

97 CTAAAGCAAGATAGAA CCCTTCTGAATCGTCT

98 GCCAACAGTCACCTTG CTGAACCTGTTGGCAA

99 GAAATGGATTATTTAC ATTGGCAGACATTCTG

100 TTTT TATAAGTA TAGCCCGGCCGTCGAG

101 AGGGTTGA TTTT ATAAATCC TCATTAAATGATATTC

102 ACAAACAA TTTT AATCAGTA GCGACAGATCGATAGC

103 AGCACCGT TTTT TAAAGGTG GCAACATAGTAGAAAA

104 TACATACA TTTT GACGGGAG AATTAACTACAGGGAA

105 GCGCATTA TTTT GCTTATCC GGTATTCTAAATCAGA

106 TATAGAAG TTTT CGACAAAA GGTAAAGTAGAGAATA

107 TAAAGTAC TTTT CGCGAGAA AACTTTTTATCGCAAG

108 ACAAAGAA TTTT ATTAATTA CATTTAACACATCAAG

109 AAAACAAA TTTT TTCATCAA TATAATCCTATCAGAT

110 GATGGCAA TTTT AATCAATA TCTGGTCACAAATATC

111 AAACCCTCTTTTACCAGTAATAAAAGGGATTCACCAGTCACACGTTTT

112 CCGAAATCCGAAAATCCTGTTTGAAGCCGGAA

113 CCAGCAGGGGCAAAATCCCTTATAAAGCCGGC

114 GCATAAAGTTCCACACAACATACGAAGCGCCA

115 GCTCACAATGTAAAGC CTGGGGTGGGTTTGCC

116 TTCGCCATTGCCGGAA ACCAGGCATTAAATCA

117 GCTTCTGGTCAGGCTG CGCAACTGTGTTATCC

118 GTTAAAATTTTAACCA ATAGGAACCCGGCACC

119 AGACAGTCATTCAAAA GGGTGAGAAGCTATAT

120 AGGTAAAGAAATCACC ATCAATATAATATTTT

121 TTTCATTTGGTCAATA ACCTGTTTATATCGCG

122 TCGCAAATGGGGCGCG AGCTGAAATAATGTGT

123 TTTTAATTGCCCGAAA GACTTCAAAACACTAT

124 AAGAGGAACGAGCTTC AAAGCGAAGATACATT

125 GGAATTACTCGTTTAC CAGACGACAAAAGATT

126 GAATAAGGACGTAACA AAGCTGCTCTAAAACA

127 CCAAATCACTTGCCCT GACGAGAACGCCAAAA

128 CTCATCTTGAGGCAAA AGAATACAGTGAATTT

129 AAACGAAATGACCCCC AGCGATTATTCATTAC

130 CTTAAACATCAGCTTG CTTTCGAGCGTAACAC

131 TCGGTTTAGCTTGATA CCGATAGTCCAACCTA

132 TGAGTTTCGTCACCAG TACAAACTTAATTGTA

133 CCCCGATTTAGAGCTT GACGGGGAAATCAAAA

134 GAATAGCCGCAAGCGG TCCACGCTCCTAATGA

135 GAGTTGCACGAGATAG GGTTGAGTAAGGGAGC

136 GTGAGCTAGTTTCCTG TGTGAAATTTGGGAAG

137 TCATAGCTACTCACAT TAATTGCGCCCTGAGA

138 GGCGATCGCACTCCAG CCAGCTTTGCCATCAA

139 GAAGATCGGTGCGGGC CTCTTCGCAATCATGG

140 AAATAATTTTAAATTG TAAACGTTGATATTCA

141 GCAAATATCGCGTCTG GCCTTCCTGGCCTCAG

142 ACCGTTCTAAATGCAA TGCCTGAGAGGTGGCA

143 TATATTTTAGCTGATA AATTAATGTTGTATAA

144 TCAATTCTTTTAGTTT GACCATTACCAGACCG

145 CGAGTAGAACTAATAG TAGTAGCAAACCCTCA

146 GAAGCAAAAAAGCGGA TTGCATCAGATAAAAA

147 TCAGAAGCCTCCAACA GGTCAGGATCTGCGAA

148 CCAAAATATAATGCAG ATACATAAACACCAGA

149 CATTCAACGCGAGAGG CTTTTGCATATTATAG

150 ACGAGTAGTGACAAGA ACCGGATATACCAAGC

151 AGTAATCTTAAATTGG GCTTGAGAGAATACCA

152 GCGAAACATGCCACTA CGAAGGCATGCGCCGA

153 ATACGTAAAAGTACAA CGGAGATTTCATCAAG

154 CAATGACACTCCAAAA GGAGCCTTACAACGCC

155 AAAAAAGGACAACCAT CGCCCACGCGGGTAAA

156 TGTAGCATTCCACAGA CAGCCCTCATCTCCAA

157 GTAAAGCACTAAATCG GAACCCTAGTTGTTCC

158 AGTTTGGAGCCCTTCA CCGCCTGGTTGCGCTC

159 AGCTGATTACAAGAGT CCACTATTGAGGTGCC

160 ACTGCCCGCCGAGCTC GAATTCGTTATTACGC

161 CCCGGGTACTTTCCAG TCGGGAAACGGGCAAC

162 CAGCTGGCGGACGACG ACAGTATCGTAGCCAG

163 GTTTGAGGGAAAGGGG GATGTGCTAGAGGATC

164 CTTTCATCCCCAAAAA CAGGAAGACCGGAGAG

165 AGAAAAGCAACATTAA ATGTGAGCATCTGCCA

166 GGTAGCTAGGATAAAA ATTTTTAGTTAACATC

167 CAACGCAATTTTTGAG AGATCTACTGATAATC

168 CAATAAATACAGTTGA TTCCCAATTTAGAGAG

169 TCCATATACATACAGG CAAGGCAACTTTATTT

170 TACCTTTAAGGTCTTT ACCCTGACAAAGAAGT

171 CAAAAATCATTGCTCC TTTTGATAAGTTTCAT

172 TTTGCCAGATCAGTTG AGATTTAGTGGTTTAA

173 AAAGATTCAGGGGGTA ATAGTAAACCATAAAT

174 TTTCAACTATAGGCTG GCTGACCTTGTATCAT

175 CCAGGCGCTTAATCAT TGTGAATTACAGGTAG

176 CGCCTGATGGAAGTTT CCATTAAACATAACCG

177 TTTCATGAAAATTGTG TCGAAATCTGTACAGA

178 ATATATTCTTTTTTCA CGTTGAAAATAGTTAG

179 AATAATAAGGTCGCTG AGGCTTGCAAAGACTT

180 CGTAACGATCTAAAGT TTTGTCGTGAATTGCG

181 ACCCAAATCAAGTTTT TTGGGGTCAAAGAACG

182 TGGACTCCCTTTTCAC CAGTGAGACCTGTCGT

183 TGGTTTTTAACGTCAA AGGGCGAAGAACCATC

184 GCCAGCTGCCTGCAGG TCGACTCTGCAAGGCG

185 CTTGCATGCATTAATG AATCGGCCCGCCAGGG

186 ATTAAGTTCGCATCGT AACCGTGCGAGTAACA

187 TAGATGGGGGGTAACG CCAGGGTTGTGCCAAG

188 ACCCGTCGTCATATGT ACCCCGGTAAAGGCTA

189 CATGTCAAGATTCTCC GTGGGAACCGTTGGTG

190 TCAGGTCACTTTTGCG GGAGAAGCAGAATTAG

191 CTGTAATATTGCCTGA GAGTCTGGAAAACTAG

192 CAAAATTAAAGTACGG TGTCTGGAAGAGGTCA

193 TGCAACTAAGCAATAA AGCCTCAGTTATGACC

194 TTTTTGCGCAGAAAAC GAGAATGAATGTTTAG

195	AAACAGTTGATGGCTT AGAGCTTATTTAAATA
196	ACTGGATAACGGAACA ACATTATTACCTTATG
197	ACGAACTAGCGTCCAA TACTGCGGAATGCTTT
198	CGATTTTAGAGGACAG ATGAACGGCGCGACCT
199	CTTTGAAAAGAACTGG CTCATTATTTAATAAA
200	GCTCCATGAGAGGCTT TGAGGACTAGGGAGTT
201	ACGGCTACTTACTTAG CCGGAACGCTGACCAA
202	AAAGGCCGAAAGGAAC AACTAAAGCTTTCCAG
203	GAGAATAGCTTTTGCG GGATCGTCGGGTAGCA
204	ACGTTAGTAAATGAAT TTTCTGTAAGCGGAGT
205	TTTT CGATGGCC CACTACGTAAACCGTC
206	TATCAGGG TTTT CGGTTTGC GTATTGGGAACGCGCG
207	GGGAGAGG TTTT TGTAAAAC GACGGCCATTCCCAGT
208	CACGACGT TTTT GTAATGGG ATAGGTCAAAACGGCG
209	GATTGACC TTTT GATGAACG GTAATCGTAGCAAACA
210	AGAGAATC TTTT GGTTGTAC CAAAAACAAGCATAAA
211	GCTAAATC TTTT CTGTAGCT CAACATGTATTGCTGA
212	ATATAATG TTTT CATTGAAT CCCCCTCAAATCGTCA
213	TAAATATT TTTT GGAAGAAA AATCTACGACCAGTCA
214	GGACGTTG TTTT TCATAAGG GAACCGAAAGGCGCAG
215	ACGGTCAA TTTT GACAGCAT CGGAACGAACCCTCAG
216	CAGCGAAAA TTTT ACTTTCA ACAGTTTCTGGGATTT TGCTAAAC TTTT
Loop1	Loop1 AACATCACTTGCCTGAGTAGAAGAACT
Loop2	Loop2 TGTAGCAATACTTCTTTGATTAGTAAT
Loop3	Loop3 AGTCTGTCCATCACGCAAATTAACCGT
Loop4	Loop4 ATAATCAGTGAGGCCACCGAGTAAAAG
Loop5	Loop5 ACGCCAGAATCCTGAGAAGTGTTTTT
Loop6	Loop6 TTAAAGGGATTTTAGACAGGAACGGT
Loop7	Loop7 AGAGCGGGAGCTAAACAGGAGGCCGA
Loop8	Loop8 TATAACGTGCTTTCCTCGTTAGAATC
Loop9	Loop9 GTACTATGGTTGCTTTGACGAGCACG
Loop10	Loop10 GCGCTTAATGCGCCGCTACAGGGCGC

Supplementary Table S2

Sequences of aptamer-staples: Numbering refers to staple strand number. "Edge" or "Middle" refers to the position of the aptamer modification within the staple sequence. Underlined residues indicate the aptamer sequence. Non-underlined residues are staple sequences. See Figure S14 for schematic. NA = not applicable as no binding is observed.

Aptamer ID	Sequence	K_d (nM)
**11 Edge**	CCTAATTTACGCTAACGAGCGTCTAATCAATA CTGGGCGGTAGAACCATAGTGACCCAG	596 +/- 107
**87 Middle**	TCATTACCCGACAATACTGGGCGGTAGAACCATAGTGACCCAG AACAACATATTTAGGC	1725 +/- 244
**111 Edge**	AAACCCTCTTTTACCAGTAATAAAAGGGATTCACCAGTCACACGTTTT CTGGGCGGTAGAACCATAGTGACCCAG	606 +/- 164
**121 Middle**	TTTCATTTGGTCAATACTGGGCGGTAGAACCATAGTGACCCAGACCTGTTTATATCGCG	NA
**211 Edge**	GCTAAATCTTTTCTGTAGCTCAACATGTATTGCTGACTGGGCGGTAGAACCATAGTGACCCAG	355 +/- 73
